# Pollination and plant reproduction in the Cerrado, the world's most biodiverse savanna

**DOI:** 10.1111/brv.70073

**Published:** 2025-09-16

**Authors:** João C. F. Cardoso, Renata Trevizan, Pietro K. Maruyama, Ana P. S. Caetano, Rogério V. Gonçalves, Yasmine Antonini, Paulo E. Oliveira

**Affiliations:** ^1^ Programa de Pós‐Graduação em Ecologia, Conservação e Manejo da Fauna Silvestre, Universidade Federal de Minas Gerais Belo Horizonte Minas Gerais 31270‐901 Brazil; ^2^ Centro de Síntese Ecológica e Conservação, Departamento de Genética, Ecologia e Evolução – Instituto de Ciências Biológicas Universidade Federal de Minas Gerais Belo Horizonte Minas Gerais 31270‐901 Brazil; ^3^ Instituto de Biociências, Universidade Federal de Mato Grosso Cuiabá Mato Grosso 78060900 Brazil; ^4^ School of Science, Environmental Futures Research Centre University of Wollongong Wollongong 2522 New South Wales Australia; ^5^ Departamento de Biodiversidade, Evolução e Meio Ambiente Universidade Federal de Ouro Preto Ouro Preto Minas Gerais 35400‐000 Brazil; ^6^ Instituto de Biologia Universidade Federal de Uberlândia Uberlândia Minas Gerais 38405‐320 Brazil

**Keywords:** Brazilian neotropical savanna, flowering plants, plant–pollinator interactions, phenology, fire, habitat complementarity

## Abstract

The Brazilian Cerrado is a continental‐wide biodiversity hotspot and the most species‐rich savanna ecosystem in the world. The main aspect characterising this biodiversity is that the landscape is arranged as an intricate mosaic of different plant formations, including grasslands, savannas, and forests, each harbouring distinct but interconnected communities. Seasonality and natural fires are key and ancient natural factors in the biome, with organisms showing many adaptations. The Cerrado is also home to millions of people, and the essential ecosystem services provided for agricultural production make it one of the world's major crop regions. However, it has undergone intense destruction in the last decades, with conservation concerns historically overshadowed by the neighbouring Amazonia and Atlantic Forest biomes. Considering the importance of pollination and plant reproduction for maintaining terrestrial ecosystems, we synthesise the known information for the Cerrado as an illustrative example that could be applied to other megadiverse ecosystems worldwide. Although apomixis (asexual seed formation) and self‐pollination mechanisms occur to a lesser extent, most plants in the Cerrado require biotic pollination. For instance, this is the case for some dioecious and monoecious species. However, the majority of plants have bisexual flowers, with the frequency of self‐incompatibility increasing towards denser plant formations such as forests, illustrating differences in dependency on pollination across habitats. Many Cerrado plants adopt strategies favouring outcrossing, including distyly, enantiostyly, heteranthery, and dichogamy. Although plant–pollinator interaction networks are mostly generalised, the pollinators are organised into guilds, with bees pollinating most plants and using several resources. Other common guilds include beetles, moths, hummingbirds, and bats. Importantly, flowering phenology peaks across plant formations at different times of the year, creating habitat complementarity across the vegetation mosaic that continuously sustains transiting pollinators. Thus, the interaction between plants and pollinators connects and is sustained by landscape complexity, which should be regarded as essential for ecosystem conservation. In this context, periodic fires that trigger massive flowering and promote biomass reduction are an essential natural disturbance that maintains the diversity of open landscapes. The interdependence of plants and pollinators in the face of the ongoing destruction of the Cerrado adds another challenge for its conservation, and highlights the necessity for conserving complementary habitats at the landscape level. While forest formations are granted protection by law, these alone are insufficient to maintain high pollinator diversity, with potential cascading effects on the ecosystem services they provide and requiring the maintenance of the neglected grasslands and savannas. Thus, the simultaneous conservation and restoration of the mosaic plant formations across the landscape will be crucial for the future of the Cerrado.

## I. INTRODUCTION

Even though the greatest diversity of flowering plants is found in tropical forests, open tropical ecosystems around the globe also harbour a considerable portion of Earth's biodiversity (Solbrig, Medina & Silva, 1996; Overbeck *et al*., 2015). These often‐neglected environments, including tropical savannas, are seen as less charismatic and have historically received less attention from researchers, funding agencies, conservation initiatives, and the public (Overbeck *et al*., 2015; Silveira *et al*., 2022; Bispo *et al*., 2024). However, tropical savannas cover over 20% of the planet's land surface and contribute to approximately one‐third of global primary productivity (Grace *et al*., 2006). At the same time, these areas are home to *ca*. 20% of the human population and comprise most of the active regions of agriculture and livestock farming in the world, thus being important ecologically, economically, and culturally (Solbrig *et al*., 1996).

**Fig. 1:**
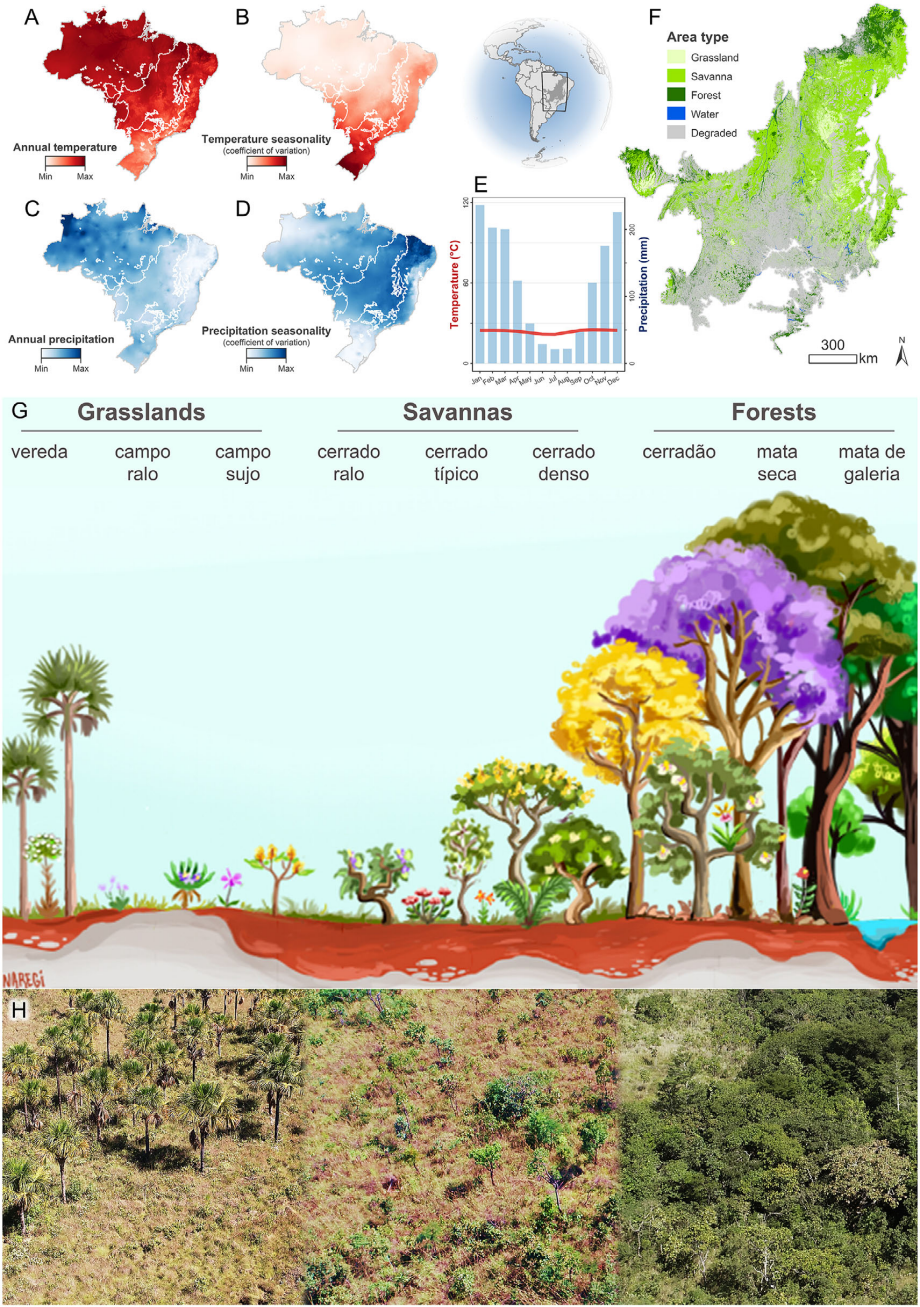
Cerrado biome location and characteristics. (A–D) Mean annual temperature, temperature seasonality, mean annual precipitation, and precipitation seasonality of Brazil, emphasising the Cerrado (shaded region in the inset map). (E) Walter and Lieth's climate diagram for the Cerrado biome demonstrating the marked seasonality. The red line and blue bars represent the monthly temperature and monthly precipitation, respectively. (F) Land cover of the Cerrado indicating areas of grassland (5.1%), savanna (31.8%), forest (14.8%), water (0.7%), and degraded landscape (44.9%). (G) Illustration depicting the diversity of plant formations including grasslands [vereda; campo ralo (Portuguese for ‘open grassland’); campo sujo (‘dense grassland’)], savannas/cerrado *sensu stricto* [cerrado ralo (‘open cerrado *s.s*.’); cerrado típico (‘typical cerrado *s.s*.’); cerrado denso (‘dense cerrado *s.s*.’)] and forests [cerradão; mata seca (‘dry forest’); mata de galeria (‘gallery forest’)]. Artwork credit: Giovana Narezi. (H) Continuum demonstrating the spatial change of plant formations (compilation of three different photographs).

In Brazil, the Cerrado biome is a phytogeographic domain comprising tropical savanna with certain morphoclimatic characteristics (Fig. 1). Occupying an impressive 26.2% of the country's territory with an area of 2,230,841 km^2^, the Cerrado is the second‐largest biome in South America and home to millions of people (Vieira *et al*., 2022). It is considered a biodiversity hotspot due to its high species richness and endemism (Myers *et al*., 2000; Strassburg *et al*., 2017; Vieira *et al*., 2022). It is regarded as the world's most biodiverse savanna ecosystem, harbouring 12,180 species of angiosperms catalogued to date, with 7,395 being Brazilian endemics (Forzza *et al*., 2012; Reflora, 2024). These comprise 3.5% of all vascular plants worldwide within only 0.4% of the planet's land surface (Freiberg *et al*., 2020; Silva *et al*., 2024). One of the factors explaining this exceptional diversity is the heterogeneity of plant formation types that harbour distinct communities across a landscape mosaic (Eiten, 1972; Ribeiro & Walter, 2008; Borghetti *et al*., 2019). Its large extension also contributes to species richness since there is a high β‐diversity across areas at a regional scale (Ratter, Bridgewater & Ribeiro, 2003; Bridgewater, Ratter & Ribeiro, 2004). Finally, since the Cerrado is located in a central portion of the continent, its diversity is also increased by incorporating floristic elements from other neighbouring biomes, including the Atlantic and Amazon forests, Caatinga, Pantanal, and Chaco (Eiten, 1972; Oliveira‐Filho & Ratter, 1995; Simon *et al*., 2009; Borghetti *et al*., 2019).

The Cerrado was poorly known regarding its characteristics and biodiversity by the time that Eiten (1972) published a seminal review about the biome. However, agricultural expansion has intensified over the Cerrado since then, becoming the main agricultural frontier in Brazil and currently one of the world's major crop regions (Strassburg *et al*., 2017; Vieira *et al*., 2022; Salatino & Salatino, 2023). Vast and easily manageable flatlands at low cost, new soil fertility correction techniques, no‐till farming methods, and new crop varieties enabled the Cerrado to become an ‘agricultural Eldorado’ (Salatino & Salatino, 2023). However, this economic growth has led to intense environmental change and biodiversity loss (Strassburg *et al*., 2017; Salatino & Salatino, 2023). In the last 50 years, the biome has been occupied and destroyed at a much faster rate than most other tropical plant formations, with *ca*. 45% of its area already degraded (Fig. 1F). It is a poorly legally protected territory, being historically overshadowed by its neighbouring biomes such as Amazonia and the Atlantic Forest (Shirai *et al*., 2024). In addition, open plant formations of the Cerrado mosaic are neglected and viewed as less important for conservation, despite harbouring a unique biodiversity (Overbeck *et al*., 2015; Silveira *et al*., 2022; Bispo *et al*., 2024). If the current destruction rate continues, anthropic activities will lead to a massive loss of biodiversity as well as of ecosystem services provided by this biome, including water resources, hydroelectric power generation, and sustainable agriculture (Strassburg *et al*., 2017; Klink *et al*., 2020; Vieira *et al*., 2022; Salatino & Salatino, 2023).

Flowering plants represent *ca*. 90% of all plant species and largely dominate terrestrial ecosystems, especially in tropical regions (Sauquet, Ramírez‐Barahona & Magallón, 2022). Their interactions with animals are widespread and frequently interdependent: the flowers attract and usually reward animals that, in turn, ensure sexual reproduction and genetic diversity by transporting pollen over long distances (Barrett, 2002; Ollerton, Winfree & Tarrant, 2011; Ollerton, 2017; Tong *et al*., 2023). Approximately 94% of tropical plants depend on biotic vectors for reproduction (Ollerton *et al*., 2011). As in other tropical areas, most plants in the Cerrado depend on animal pollination to complete their reproductive cycle. Therefore, pollinators are vital for the sexual reproduction of plants in this biome (Oliveira & Gibbs, 2000; Barbosa & Sazima, 2008). Although the Cerrado flora has been extensively studied and is now better known than in Eiten's time (see Forzza *et al*., 2012; Reflora, 2024), suitable data to inform plant and pollinator conservation strategies and public policies are still missing. Greater awareness has emerged about the key role of biotic pollination in maintaining biodiversity and ecosystem functioning (Ollerton, 2021; Rodger *et al*., 2021; Wei *et al*., 2021; Cardoso *et al*., 2023b; Tong *et al*., 2023) as well as providing ecosystem services such as crop pollination that support food production and economic value (Oliveira *et al*., 2024b). Thus, conserving and restoring plant–pollinator interactions in the Cerrado is mandatory to preserve plant populations and vegetation structure, maintaining high levels of diversity.

Such knowledge may be especially relevant considering the importance of the Cerrado as one of the major agricultural producers in the world and the importance of wild pollinators in providing ecosystem services essential for food production (Oliveira *et al*., 2024b). The Cerrado harbours numerous cultivated and native plants that rely on biotic vectors, underscoring the economic and sociocultural importance of pollination (Giannini *et al*., 2015; Oliveira *et al*., 2024b). For instance, while the Cerrado is one of the most productive soybean regions, accounting for *ca*. 15% of global production (TNC, 2019; Oliveira *et al*., 2024b), this crop is dependent on pollination services, especially in low‐ to mid‐latitudes such as those of the Cerrado (Cunha *et al*., 2023).

To understand comprehensively pollination processes in a megadiverse biome, the high biodiversity must be associated with sufficient knowledge. The Cerrado is one of the few ecosystems in the world that meet these criteria. In addition to being the most biodiverse savanna, there is a wealth of knowledge generated by past research, and it is one of the best‐studied biomes in Brazil regarding pollination (Valadão‐Mendes *et al*., 2025). In contrast to mostly forested ecosystems, where pollination often occurs at the canopy and research is logistically challenging, many Cerrado flowers are at an accessible height, allowing for more feasible observational and experimental studies. However, the wealth of information is scattered and needs to be comprehensively reviewed. Furthermore, it is necessary to define knowledge gaps and future research directions that foster conservation and management practices in this unique biome. Considering the importance of the Cerrado for global biodiversity, the essential ecosystem services provided by pollination, and the urgency for conservation strategies, we review herein the reproductive biology of angiosperms and their interaction with pollinators in the Cerrado.

We combine a narrative and systematic review approach (see online Supporting Information, Appendix S1) to understand the reproductive strategies of Cerrado plants, the pollinators responsible for plant reproduction, the interaction networks between them, and how spatiotemporal changes influence pollination, including seasonality, fire, and the mosaic arrangement of plant formations. Thus, we aim to integrate knowledge on plants and pollinators with the intrinsic historical and landscape characteristics of the Cerrado, to enable the functioning of pollination to be understood comprehensively at the biome level. By highlighting the mosaic nature and interconnected functioning of Cerrado habitats, we demonstrate that the integrated conservation of the various plant formations at the landscape scale is essential to maintaining sustainable pollination services and plant reproduction in the Cerrado. We use our assessment of pollination in the Cerrado as a starting point to establish future research guidelines addressing both theoretical and practical issues. Understanding how plants and pollinators interact on the macroscale of the biome will help predict, mitigate, and hopefully avoid the effects of ongoing anthropogenic changes.

## II. ENVIRONMENTAL CHARACTERISTICS

The Cerrado is characterised by high temperatures, with a mean monthly temperature of 23.8 °C (Fig. 1A, E; Appendix S2). Precipitation rates are also high, with a mean annual precipitation of 1,462 mm (Fig. 1C, E). Temperature and precipitation show a marked seasonality (Fig. 1B, D, E) with hot and wet summers (October–March) and hot to mild temperatures during dry winters (April–September). This set of specific bioclimatic variables determines the biome, leading to its unique characteristics that are distinctive from nearby biomes. In addition to seasonality, these include natural fires at the end of the dry season, with organisms adapted to these millennial conditions (Simon *et al*., 2009; Borghetti *et al*., 2019).

The plant formations of the Cerrado comprise varied grasslands, savannas, and forests, with either dry or wet soils (Fig. 1F–H; see the 14 main types in Ribeiro & Walter, 2008). These structural forms of plant communities constitute an intricate mosaic in the landscape. Savanna formations are generally called ‘cerrado *sensu stricto*’ (not capitalised; hereafter cerrado *s.s*.), originally the most common vegetation type covering *ca*. 70% of the biome (Fig. 1G, Eiten, 1972; Borghetti *et al*., 2019). These may be further divided into open cerrado *s.s*., typical cerrado *s.s*., and dense cerrado *s.s*. according to the continuum of woody plant coverage (Fig. 1G). When the canopy of a dense cerrado *s.s*. becomes taller and continuous, this constitutes a forest formation called cerradão. Other forest areas include evergreen and semideciduous dry forests and riparian and gallery forests associated with water courses. Grasslands include several formations, from flatlands to the campos rupestres (rupestrian fields) in rocky outcrops. The Cerrado also harbours seasonally flooded areas, such as some gallery forests, riparian forests, and wet grasslands (Ribeiro & Walter, 2008; Borghetti *et al*., 2019). One of the most common and important wetlands is the veredas swamps, which occur in enclosed valleys where the water table emerges.

## III. REPRODUCTIVE BIOLOGY

### (1) Asexual reproduction

Not all Cerrado plants reproduce sexually. Some can reproduce asexually through vegetative growth, such as budding and resprouting, especially after fires (Pilon *et al*., 2021). Asexual reproduction involving seed formation also exists. This is the case for apomixis, a mechanism in which asexual embryos are formed regardless of fertilisation (Hojsgaard & Hörandl, 2019). Apomixis commonly results in the formation of clones or less genetically variable embryos than those with a sexual origin (Hojsgaard & Pullaiah, 2022).

We reviewed the presence of apomixis for the Cerrado and found that it has been identified in 56 species to date (Table S1; Appendix S1). Based on the 12,180 angiosperm species catalogued in the Cerrado, this represents 0.5% of the flora. However, this is likely an underestimate because most plants have not yet been investigated, particularly those from taxa traditionally known to exhibit this system. Thus, this and the following calculations based on the Cerrado flora (in the next topics) should be interpreted with caution. Apomixis was widely distributed across 20 families, with the most common ones being Melastomataceae and Bignoniaceae, with 19 and 8 species, respectively (Table S1). Determining if a plant is apomictic is a laborious process that involves controlled pollination treatments, anatomical analyses to observe ovule and seed development, or flow cytometric seed screening. Therefore, data on the distribution of apomixis within communities is still limited. Although biased by the selected species, some subsamples related to plant habit and specific taxonomic groups indicate varying proportions of apomictic species. For example, they may constitute between 5% and 7% of woody plant communities from cerrado *s.s*., mostly composed of Melastomataceae species (Saraiva, Cesar & Monteiro, 1996; Oliveira & Gibbs, 2000). On the other hand, within‐family assessments show that apomixis in Melastomataceae communities from rupestrian field and cerrado *s.s*. areas can reach 10% and 64%, respectively (Goldenberg & Shepherd, 1998; Santos *et al*., 2012). This proportion varies according to the identity of the sampled groups, such as the case of the Miconieae tribe, in which apomixis is known to be more frequent (Caetano & Oliveira, 2022).

Apomictic plants seldom produce clones exclusively, as apomixis is mostly facultative and retains sexuality to a certain extent (Hojsgaard & Pullaiah, 2022). In a few species, such as in certain Melastomataceae, sexual and apomictic embryo sacs co‐occur even in the same ovule (Caetano & Oliveira, 2022). In other cases involving sporophytic apomixis, such as in some Bignoniaceae and Malvaceae species, the formation of adventitious embryos commonly depends on an endosperm formed after fertilisation so that apomictic and sexual embryos coexist in the same seed (Mendes‐Rodrigues *et al*., 2005; Sampaio, Bittencourt Júnior & Oliveira, 2013). Furthermore, other processes, such as mutation and recombination during anomalous or incomplete meiosis, may generate variability in apomictic populations (Hörandl & Paun, 2007). A typical example of this situation occurs in *Miconia albicans* (Melastomataceae), a treelet whose individuals are obligatorily apomictic and do not produce viable pollen grains due to the degeneration of meiotic products (Caetano *et al*., 2013). Given its reproductive autonomy, rapid growth, and high plasticity, this is a pioneer species and often locally dominant, being one of the most widely distributed Cerrado woody plants (Ratter *et al*., 2003). It has been found that most adults and seedlings are not clones and show relatively high genetic diversity among individuals, which is probably generated by restitutional meiosis (Dias *et al*., 2021). This variability in apomictic species may be important to guarantee adaptability to the heterogeneous environments of the Cerrado.

Apomixis may be associated with polyembryony (i.e. the presence of more than one embryo per seed), although other processes can also lead to the formation of polyembryonic seeds (Hojsgaard & Pullaiah, 2022). This phenomenon primarily occurs in apomictic plants due to the development of multiple adventitious embryos (i.e. originating from nucellar or integumentary cells) within a single seed. These adventitious embryos may also be associated with a sexually derived embryo (Hojsgaard & Pullaiah, 2022). Polyembryonic seeds with both sexual and adventitious embryos have been reported in some Cerrado species of Bignoniaceae, Malvaceae, and Orchidaceae (Sampaio *et al*., 2013; Mendes‐Rodrigues *et al*., 2019; Costa *et al*., 2024). In Melastomataceae, in addition to adventitious embryos, the concurrent development of both apomictic and sexual embryo sacs within a single ovule can also contribute to forming polyembryonic seeds (Caetano & Oliveira, 2022).

Polyembryony may involve disadvantages such as competition between embryos that potentially reduces the conversion of embryos into seedlings (Shaanker & Ganeshaiah, 1997; Mendes‐Rodrigues *et al*., 2005, 2012). However, it allows the coexistence of sexual and asexual embryos within the same seed, increasing the chances of survival, germination, and fitness of at least one embryo (Mendes‐Rodrigues *et al*., 2012). Moreover, apomixis and polyembryony can function as compensatory mechanisms for problems during sexual reproduction and the reduced number of seeds per fruit (Shaanker & Ganeshaiah, 1997). Therefore, apomixis and polyembryony have been interpreted as strategies related to unpredictable environments limited by water availability and nutrients (Marinho *et al*., 2023).

The reproductive assurance provided by apomixis and polyembryony also offers better colonising abilities, such as in the aforementioned *M. albicans*. Apomictic plants are widely distributed in the Cerrado region. These plants typically combine polyploidy, hybrid origin, and uniparental reproduction, which collectively contribute to their larger distribution ranges compared to their sexual relatives (Hörandl, 2009). The range expansion of apomictic lineages has been termed geographic parthenogenesis (Hörandl, 2009) and has been observed in some Cerrado plants, such as *Eriotheca* (Malvaceae; Mendes‐Rodrigues *et al*., 2019), *Miconia* and *Microlicia* (Melastomataceae; Santos *et al*., 2012; Caetano & Oliveira, 2022), and *Zygopetalum* (Orchidaceae; Costa *et al*., 2024).

It is important to emphasise that apomictic plants may be entirely independent of pollination to produce viable seeds (autonomous apomixis; Hojsgaard & Pullaiah, 2022). Most cases of apomixis in Cerrado plants are detected through pollen exclusion or emasculation experiments, indicating autonomous apomixis (41 species; Table S1). However, pseudogamous species rely on pollination to ensure asexual embryo development (Hojsgaard & Pullaiah, 2022). Since pseudogamy is more difficult to detect, this process has been recorded in only 12 species and is likely underestimated in Cerrado plants (Table S1). Fertilisation ensures endosperm formation, except in *Zygopetalum*, in which it stimulates ovule development (Table S1). Despite depending on pollination, pseudogamous plants are self‐fertile and exhibit advantages associated with uniparental reproduction, such as enhanced colonising abilities.

### (2) Autonomous reproduction

**Fig. 2:**
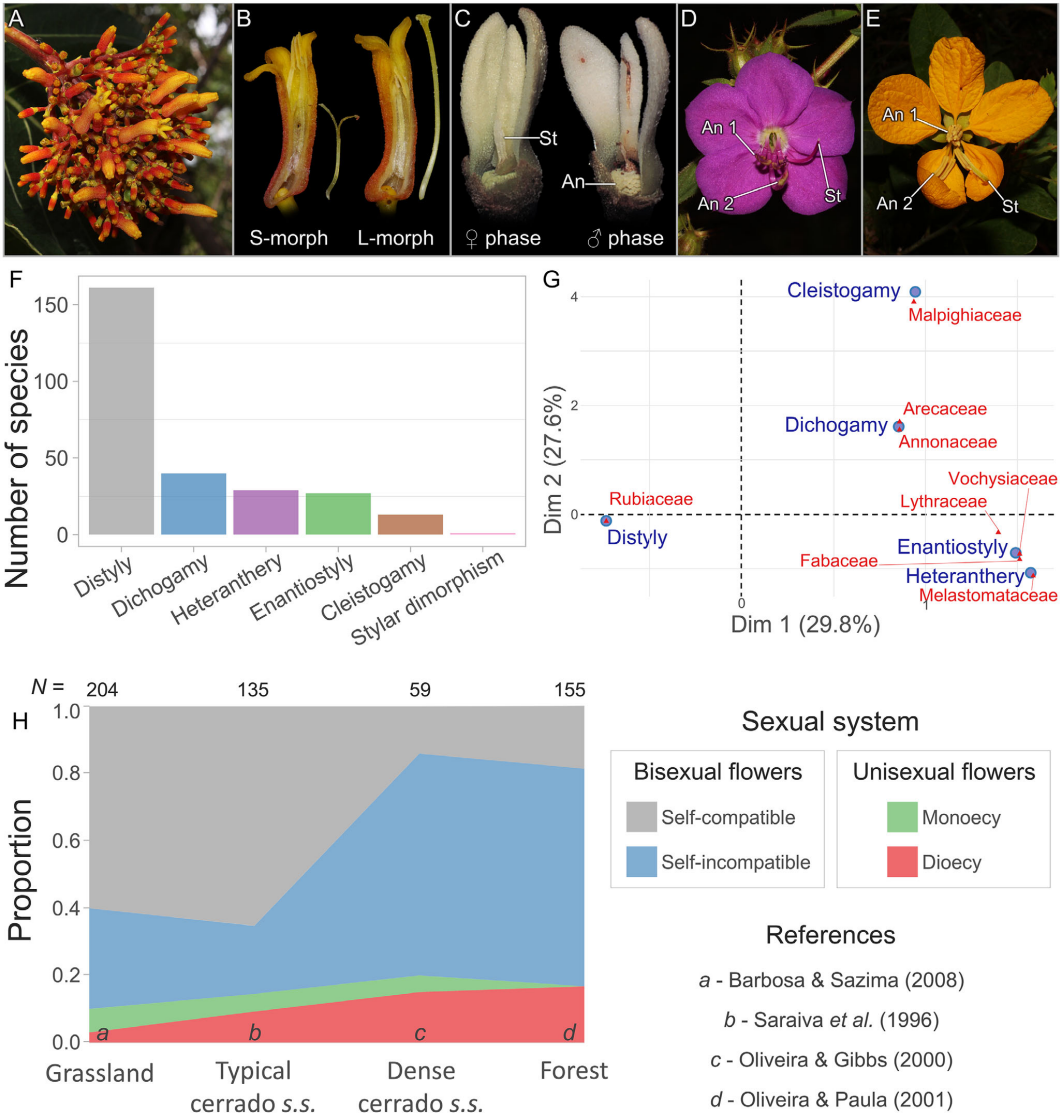
Cerrado floral and sexual systems. (A) The common *Palicourea rigida* (Rubiaceae) is an example of distyly showing (B) the short‐styled (S‐) and long‐styled (L‐) morphs, which have reproductive organs reciprocally positioned (stigmas placed outside). (C) *Xylopia emarginata* (Annonaceae) is an example of dichogamy of the type protogyny, where the female (♀) phase starts before the male (♂) phase, and these usually do not overlap. (D) *Rhynchanthera grandiflora* (Melastomataceae) and (E) *Senna velutina* (Fabaceae) are examples of both enantiostyly and heteranthery. In enantiostyly, the stigma (St) (and sometimes anthers) is laterally deflected in relation to the central axis of the flower. In heteranthery, there is the differentiation of stamens within the same flower in terms of morphology, spatial arrangement and sometimes colour. An 1 and An 2 represent smaller (feeding) and larger (pollinating) anthers. (F) Number of species in the Cerrado reported as having each of the floral systems surveyed. (G) Correspondence analysis (CA) biplot showing the relationship between floral systems and the main plant families along the first two dimensions (Dim 1 and Dim 2). Contributions are expressed in percentages within parentheses. The closer the occurrence in the biplot, the stronger the relationship. (H) Sexual and incompatibility systems in different plant formations. References from which data were extracted are represented by letters at the bottom of the plot. The sample size for each study is shown above the plot (Oliveira & Paula, 2001).

Some sexual plants rely on autogamy to assure seed formation and independence of pollen flow (Barrett, 2002). Autonomous self‐pollination occurs when the pollen of a flower is transferred from the anthers (male structure) and deposited onto the stigmas (female structure) of the same flower without the action of any external pollinating agents (Sicard & Lenhard, 2011; Cardoso *et al*., 2018). For instance, this can occur in plants with bisexual flowers, i.e. with both anthers and stigmas, by reducing the temporal and spatial separation between the sexual organs, allowing self‐pollen deposition (Sicard & Lenhard, 2011). Certain herbaceous plants can produce fruits and seeds by cleistogamy, a specialised floral system in which flowers never open and self‐deposit pollen onto stigmas, functioning as a reproductive assurance mechanism (Cardoso *et al*., 2018). Cleistogamous flowers usually co‐occur with chasmogamous flowers, i.e. that open normally, allowing outcrossing to occur in parallel with self‐pollination. We reviewed the cleistogamy system for the Cerrado and found that although it occurs, it is uncommon, especially when compared to cross‐pollination strategies (Fig. 2F; Appendix S1). It has been reported in 13 species from Acanthaceae, Apocynaceae, Lentibulariaceae, Malpighiaceae, and Mayacaceae (Fig. 2G; Table S2; Appendix S1). Albeit underestimated, this represents only 0.1% of the Cerrado angiosperm flora (12,180 spp.). Notably, a self‐pollination strategy seems less common even in the herbaceous flora (Barbosa & Sazima, 2008).

**Fig. 3:**
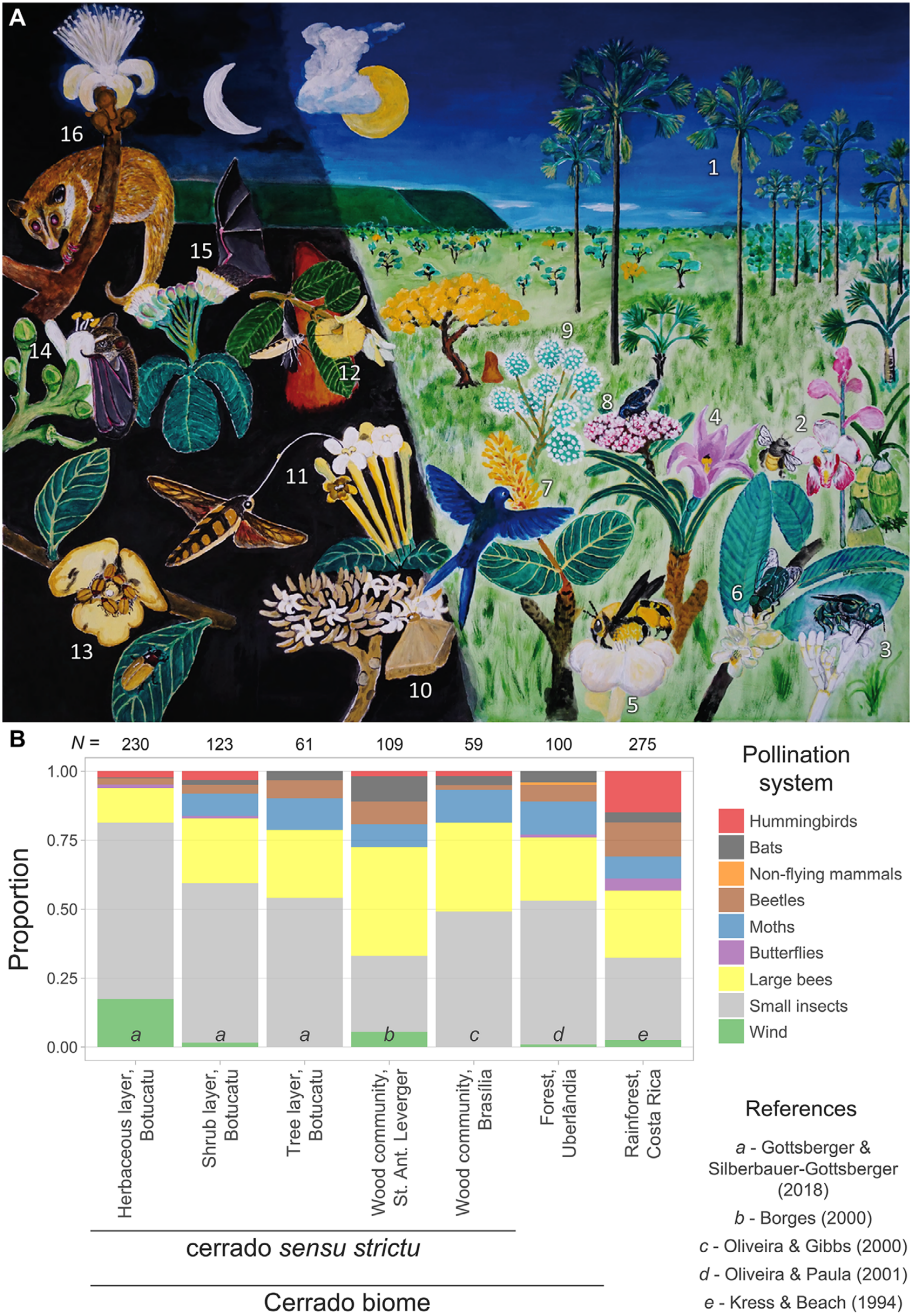
Pollination systems in plants of the Cerrado. (A) Illustration of the diversity of pollination systems (represented by numbers). Many plants are totally or partially wind‐pollinated, such as the buriti palm (*Mauritia flexuosa* – Arecaceae), typical of vereda wetlands that can also be pollinated by small insects (1). Grasses and other herbaceous plants are also wind‐pollinated. However, plants are predominantly pollinated by bees. Some do not offer any reward, such as plants of the genus *Cyrtopodium* (Orchidaceae), which are pollinated by large (Centridini) bees that are deceived (2). Other plants have different types of reward, such as nectar in *Psychotria* spp. (Rubiaceae) which can attract small bees such as those of the Euglossini tribe (3), or pollen as in some *Vellozia* spp. (Vochysiaceae) that can attract eusocial small bees (4). Solitary bees are important pollinators of reward plants, such as *Centris* spp. in *Eriotheca pubescens* (Malvaceae) (5). Flies of various groups share the pollination of many plants with small bees, such as in *Casearia grandiflora* (Salicaceae) (6). Hummingbirds are specialised pollinators that feed exclusively on nectar, such as in the distylous *Palicourea rigida* (Rubiaceae) (7). Passeriformes seldom visit flowers, although they seem to be the most important pollinators of *Hortia brasiliana* (Rutaceae) (8). Some other plants are pollinated by other small insects, including ants in the case of some Eriocaulaceae (9). Several plants depend on nocturnal pollinators, such as *Aspidosperma macrocarpum* (Apocynaceae), pollinated by small moths from different groups that land on flowers searching for nectar (10). Large moths, such as those of the Sphingidae family, are important and specialised pollinators that visit tubular flowers in mid‐flight and transfer pollen over long distances. They are the main pollinators of *Tocoyena formosa* (Rubiaceae) (11) and *Qualea grandiflora* (Vochysiaceae) (12). Beetles constitute another common group of nocturnal visitors. For instance, the common *Annona* spp. (Annonaceae) are mainly pollinated by *Cyclocephala* spp., a specialised group of beetles (Scarabeidae ‐ Dynastinae) (13). Bats are also important nocturnal pollinators, with some being more opportunistic, such as *Carollia* sp. in *Hymenaea stilbocarpa* (Fabaceae) (14). Other bats, such as *Glossophaga* spp., are more specialised and hover in front of flowers (like hummingbirds and long‐tongued moths) to feed on nectar. This is the case of *Caryocar brasiliense* (Caryocaraceae) (15). Albeit rare, non‐flying mammals can visit and even pollinate some flowers, such as *Caluromys lanatus* on *Pseudobombax tomentosum* (Malvaceae) (16). Artwork credit: Paulo E. Oliveira. (B) Frequency of the main pollination systems in different Cerrado areas from distinct localities and comparison with a tropical rainforest. To allow comparison among studies, generalised insect pollination systems that included wasps, flies, and other small bees are grouped as ‘small insects’. Herbaceous (<1 m), shrub (≥1 m and <3 m), and tree (≥3 m) represent the layer classes of the same plant community. References from which data were derived are represented by letters at the bottom of the plot. The sample size for each study is shown above the plot.

Overall, asexual and autonomous reproduction increases the success of seed formation, leads to the uniparental origin of populations, and increases the chances of persistence and colonisation of new areas after periods of environmental limitation or change, bypassing dependence on the availability of pollinators. However, studies of Cerrado plants at the community level have shown that only a small proportion of species are reproductively autonomous, and most plants are completely or mostly dependent on pollinators (Fig. 3). Below, we discuss the diverse strategies found in Cerrado plants to achieve pollinator‐mediated allogamous reproduction.

### (3) Pollinator‐dependent reproduction

Most Cerrado plants are allogamous and require pollen transport among flowers of different individuals, i.e. cross‐pollination. Several interesting floral strategies boost biotic pollination in populations, potentially leading to increased genetic variation and greater success in the heterogeneous environments of the biome. The mosaics of plant formations and the continental distribution of the Cerrado favour the adaptation of these plants to local environments with different pollinators, water availability, nutrients, luminosity, competition with other plants, and historical climatic instability (Rech *et al*., 2021). In this context, sexual reproduction provides genetic diversity and adaptability for the plants of the region. For a long time, it was thought that vegetative reproduction was more important than sexual reproduction in Cerrado plants, supposedly limited by abiotic factors associated with climate and disturbances, such as seasonality and fire, respectively (Rizzini, 1965). However, studies in the 1970s and subsequent decades showed otherwise, demonstrating that angiosperms reproduce *via* seeds. Furthermore, an increasing number of pollination investigations have indicated that the majority of plants (> 80%) require biotic pollination to form fruits and viable seeds (Oliveira & Gibbs, 2002; Barbosa & Sazima, 2008), a pattern that has seemingly persisted for a long time. For instance, a study on fossil pollen records indicated that biotic pollination has dominated the biome for at least 15,000 years since 93% of the recorded pollen taxa potentially required some animal vector (Escobar‐Torrez, Cassino & Ledru, 2024). Such pollinators promote allogamy and ensure genetic variability since they transport pollen over long distances, sometimes more than several kilometers (Borges *et al*., 2020). As discussed below, Cerrado plants rely on morpho‐physiological mechanisms to ensure pollen flow and successful reproduction.

#### (a) Sexual and incompatibility systems

The arrangement of sexual functions within and across flowers and individuals is an important element of plant reproduction. There is a great diversity of sexual systems in Cerrado plants, reflecting dependence on pollen transport by biotic vectors. We gathered data from the literature on the distribution of flower sexuality (*sensu* Cardoso *et al*., 2018) within species in different areas to understand plant gender expression in the biome and how it varies across plant formations (Fig. 2H). We also considered studies that tested the self‐incompatibility of plants in the community to understand the pressure for crossing in bisexual flowers.

Dioecy is an extreme example of how plants may assure cross‐pollination. In this system, populations have individuals with a single type of unisexual flower, either female or male (Barrett & Hough, 2013; Cardoso *et al*., 2018). Thus, reproduction is only achieved if pollen from male plants is effectively transported to females (Barrett & Hough, 2013). Cerrado dioecious plants usually have a generalist morphology and are pollinated by small insects (Oliveira, 1996a). Based on available studies, we found that, on average, 11% of the Cerrado plants were dioecious. The proportion of dioecy increases towards more closed plant formations. Grassland, typical cerrado *s.s*., dense cerrado *s.s*., and forest showed 3%, 9%, 15%, and 17% of dioecious plants, respectively (Fig. 2H). Although these values seem low, they are high when considering that only *ca*. 6% of all angiosperm species are dioecious (Renner & Ricklefs, 1995), corroborating the dependence on obligatory allogamy of many flowering plants.

Another example of sexual system specialisation is monoecy, where plants have male and female unisexual flowers within the same individual. Compared to plants with bisexual flowers, this strategy reduces the chances of intrafloral interference and geitonogamy, i.e. pollen transfer between different flowers of the same plant (De Jong, Shmida & Thuijsman, 2008; Cardoso *et al*., 2018). The proportion of monoecious plants was lower than that of dioecious plants, with an average of 4.3%. While forests had no monoecious species, grassland, typical cerrado *s.s*., and dense cerrado *s.s*. had 7%, 5%, and 5%, respectively (Fig. 2H). Combining dioecy and monoecy, unisexual flowers comprise 15.3% of the Cerrado plant species. Although both dioecy and monoecy are often related to abiotic pollination in other parts of the world (i.e. by wind or water; Renner & Ricklefs, 1995), this is not the case for the Cerrado, especially in more closed vegetation types (Fig. 3B). This highlights the role of biotic pollination in maintaining species with strict unisexual strategies.

The presence of functional female and male reproductive organs in the same flower is an ancestral character and widespread in *ca*. 90% of angiosperms (Barrett & Hough, 2013). In agreement, Cerrado plants with bisexual flowers comprise 84.7% of species. Although bisexuality has the advantage of producing both gametophytes within the same flower, it allows self‐pollen deposition, which may lead to self‐fertilisation and result in negative impacts such as inbreeding depression (Barrett, 2002). Many plants avoid such deleterious effects through different mechanisms, including self‐incompatibility. In this case, a genetically determined biochemical reaction is responsible for rejecting self‐pollen while allowing allogamous pollen to grow (Gibbs, 2014). We found that 41% of all Cerrado plants were self‐incompatible, suggesting avoidance of autogamy. The proportion of self‐incompatibility increased towards more closed formations, being only 30% and 20% in grassland and typical cerrado *s.s*., while reaching 66% and 65% in dense cerrado *s.s*. and forest, respectively (Fig. 2H). As with the proportions of unisexual flowers, these results suggest that plants from more closed formations, composed mainly of tree species, often adopt strategies that require obligatory allogamy.

On the other hand, plants with bisexual flowers that lack a self‐incompatibility system can self‐fertilise. Overall, 39.5% of species had bisexual self‐compatible flowers (Fig. 2H). The lowest proportions are in closed environments, with dense cerrado *s.s*. and forest having 14% and 19% of bisexual self‐compatible flowers, while grassland and typical cerrado *s.s*. had 60% and 65%, respectively. These patterns may be associated with the habits of plants. Contrary to trees from closed environments that adopt obligatory allogamy, herbaceous and shrubby species from open environments have more flexible systems, which allow reproductive assurance *via* self‐pollen deposition, either mediated by pollinators or not.

#### (b) Floral systems

Despite bisexual self‐compatible plants being able to produce viable fruits and seeds after self‐pollination, they often use strategies that enhance cross‐pollination. In reality, the physical interference between sexual functions can represent a disadvantage of bisexual flowers (Barrett, 2002). In addition to inbreeding depression *via* selfing, bisexuality may reduce fitness by impairing pollen removal, which results in gamete discounting, as well as by clogging the stigma with self‐pollen, causing a barrier to deposition and growth of allogamous pollen. Many Cerrado species, either self‐compatible or not, have morphological mechanisms that reduce this physical self‐interference through spatial (herkogamy) or temporal (dichogamy) separation between sexual functions within a flower (Cardoso *et al*., 2018). These can be seen as ‘outbreeding devices’ since they increase the chances of pollen flow between different flowers and individuals (Barrett, 2002). We reviewed the floral systems for the Cerrado following the terminology introduced by Cardoso *et al*. (2018) to investigate the extent of each system and the main families involved (Table S2; Appendix S1).

Distyly was the most common floral system reported in the literature, found in 161 species, corresponding to 1.3% of the Cerrado angiosperm flora to date (Fig. 2F; Table S2). This makes the Cerrado one of the places in the world with the highest occurrence of this polymorphism. Distyly is frequently associated with the Rubiaceae (Fig. 2G; e.g. Trevizan *et al*., 2021), where it has been reported for 154 species (Table S2). Other families include Connaraceae, Erythroxylaceae, and Turneraceae (Table S2). In distylous plants, reproduction requires pollen flow between individuals with distinct floral morphs. Usually associated with a physiological incompatibility system, the floral morphs have reciprocal positioning of the sexual organs, i.e. reciprocal herkogamy (Fig. 2A, B), which promotes disassortative (intermorph) pollen transfer and increases the chances of cross‐pollination (Barrett, 2002). There were no reports of tristyly in the biome, i.e. the presence of three morphs in populations.

The second most common floral system reported for Cerrado was dichogamy, found in 40 species that correspond to 0.3% of the Cerrado flora (Fig. 2F; Table S2). It was distributed across 17 families, with the most representative being Annonaceae and Arecaceae with 18 and 6 species, respectively (Fig. 2C, G; Table S2). In dichogamy, the expression of sexual functions occurs at different times, avoiding self‐interference and boosting cross‐pollination (Barrett, 2002). Cerrado plants have examples of both types of dichogamy: protogyny (stigma receptivity before pollen release) was reported in 24 species, while protandry (pollen release before stigma receptivity) was reported in 10 species (Table S2).

Enantiostyly occurred in 27 species (0.2% of the Cerrado flora) distributed in Fabaceae, Lythraceae, Vochysiaceae, and Melastomataceae (Fig. 2D–G; Table S2). In this system, the style is laterally deflected (to the left or right) according to the central flower axis (Barrett, 2002). The morphology of enantiostylous flowers promotes pollen deposition on distinct sides of the pollinator's body, reducing self‐interference and increasing cross‐pollination (Barrett, 2002). In the Cerrado plants, right‐ and left‐styled flower types occur within the same individual (i.e. monomorphic enantiostyly; e.g. Oliveira, 1996b).

Heteranthery occurred in 29 species (0.2% of the Cerrado flora) from Fabaceae and Melastomataceae (Fig. 2D–G; Table S2). This system is characterised by the differentiation of stamens sets in the same flower in terms of morphology, spatial arrangement, size, and sometimes colour and aspects of the pollen grains (Trevizan *et al*., 2023). The adaptive explanation for heteranthery is that it allows labour division between stamen types in flowers that only offer pollen as a reward for pollinators, i.e. pollen flowers. One set of stamens is specialised in providing pollen as food for pollinators, i.e. feeding anthers, while the other produces pollen for reproduction, i.e. pollinating anthers (Barrett, 2021). This mechanism reduces interference and allows resource allocation for successful cross‐pollination. For the Cerrado plants, we confirmed that enantiostyly and heteranthery are commonly found together (Fig. 2D, E, G).

Altogether, plants in this biome have a great diversity of sexual, incompatibility, and floral systems that ensure allogamous pollen transfer, and are extremely dependent on pollination agents, whose identities we discuss below. Notably, while these distinct systems reflect the diversity of plants and their interactions with pollinators, the specific number of cases reported does not directly indicate their representativeness since this will also reflect the interest of researchers over the years in particular study topics/species.

## IV. POLLINATION SYSTEMS

Many of the recorded sexual, incompatibility, and floral systems imply that Cerrado plants are dependent on pollinators because reproduction only takes place if pollen is carried between different flowers and individuals. Since animals pollinate most plants, attraction mechanisms are fundamental. These involve various traits that exploit different sensory capabilities of pollinators, including specific scents, colours, and floral morphologies (Tandon, Koul & Shivanna, 2020). In addition, the availability of rewards in flowers stimulates visits and promotes the fidelity of pollinators, increasing the chances of future visits to different flowers. In the Cerrado, these rewards vary and can include nectar, pollen, oil, fragrances, floral parts, and even shelter for various visitors (Fig. 3A). However, some flowers do not offer resources, attracting pollinators through floral mimicry strategies (Fig. 3A; Maciel, Cardoso & Oliveira, 2020; Cardoso *et al*., 2023a,*b*).

Most plants in the biome are entomophilous [i.e. pollinated by insects (Martello *et al*., 2023; Escobar‐Torrez *et al*., 2024; Luna *et al*., 2024)]. Some species tend to be very generalist, such as *Mabea fistulifera* (Euphorbiaceae), which is visited by small insects, bats, perching birds, and even non‐flying mammals that exploit nectar and/or pollen (Vieira & Carvalho‐Okano, 1996; Olmos & Boulhosa, 2013). On the other hand, some cases involve high phenotypic and ecological specialisation, such as the case of the rewardless orchid *Phragmipedium vittatum* that mimics aphids and attracts females of two syrphid fly species looking for oviposition sites for their aphidophagous larvae (Cardoso *et al*., 2023a). However, most pollination interactions involve guilds (i.e. groups of organisms that exploit similar resources in the environment) of pollinators associated with plants bearing specific floral traits (Martins & Batalha, 2006). There is a great diversity of pollination systems in the Cerrado (Fig. 3), and the relative importance of each system varies among localities and habitats (Martello *et al*., 2023). In addition, each system has specificities that influence plant and animal communities and conservation possibilities. Understanding the impact of these peculiarities on the organisation of communities in the region requires a basic but labour‐intensive naturalistic description of the distribution of pollination systems. Here, we used available data on the distribution of pollination systems gathered for plant communities in different areas (Fig. 3B). We also included data from a Costa Rican rainforest (Kress & Beach, 1994; Appendix S3) for comparison with other tropical environments traditionally seen as models with high species richness and diversity.

As in other aspects, woody plants are better studied than herbaceous species regarding their pollination systems (Borges, 2000; Oliveira & Gibbs, 2000; Martello *et al*., 2023). Most woody plants are pollinated by diverse small insects, including mainly small bees (*sensu* Michener, 2007), which can pollinate up to *ca*. 54% of woody species in a locality (Fig. 3B; Gottsberger & Silberbauer‐Gottsberger, 2018). The second most representative group comprises large bees (*sensu* Michener, 2007), which can pollinate 39.4% of a woody plant community (Fig. 3B; Borges, 2000). Together, small insects and large bees represent the highest proportion of pollination systems across all areas. Although moths, beetles, and bats are less frequent visitors, each group can account for approximately 10% of woody species pollination, although these proportions vary among sites (Fig. 3B). Hummingbirds, butterflies, and non‐flying mammals pollinate a small proportion of woody species, sometimes being absent. Wind pollination is also underrepresented among woody plants, although at one location it represented 5.5% of the species (Borges, 2000). Compared to the Costa Rican tropical forest, the pollination guilds of woody plants appear in quite similar proportions, highlighting the diversity of mechanisms that Cerrado habitats harbour (Fig. 3B). However, there are some differences. For instance, the Cerrado has a larger proportion of pollination by small insects and large bees at some sites. Although moth and bat pollination proportions are similar, pollination by beetles, butterflies, and hummingbirds seems more common in the tropical forest.

Although woody species tend to be more common in studies of pollination guilds, Gottsberger & Silberbauer‐Gottsberger (2018) surveyed the whole environment and showed trends for other strata of the plant community. They divided plants into three layer classes based on stratification patterns: herbaceous (<1 m), shrub (≥1 m and <3 m), and tree (≥3 m) (Fig. 3B). Comparatively, one of the most obvious differences is that herbaceous plants tend to have a larger proportion of wind pollination (17.4%), given the presence of grasses and sedges (see also Monteiro *et al*., 2021). Pollination by larger bees was less frequent in the herbaceous layer, while hummingbird‐pollinated plants did not occur at the tree level. Pollination by bats and moths, both nocturnal systems, was more frequent in higher layers, including shrubs and trees. Altogether, these results show how different pollination strategies may be adopted according to plant habit and the level of stratification of the vegetation. They also highlight that generalizations about pollination systems may be biased by prevailing knowledge on the natural history of woody plants, and that other layers and plant habits require further investigation. Below, we present a synthesis of the current understanding of the main pollination systems in the Cerrado.

### (1) Abiotic pollination

Some pollination systems may involve pollen transport *via* wind, but abiotic pollination seems underrepresented (Fig. 3B; Martello *et al*., 2023). This is also the historical pattern since flowers exclusively pollinated by wind are underrepresented in fossil pollen records, accounting for only 6.9% of the pollen taxa recorded in one study (Escobar‐Torrez *et al*., 2024). Wind pollination is usually accompanied by the reduction of the perianth, producing large amounts of unclumped pollen and with spatial or temporal separation of sexes (Ackerman, 2000). Some plants are ambophilous and use both wind and insect pollination, which allows reproductive flexibility, such as some palms (Silberbauer‐Gottsberger, 1990). However, the most common groups of wind‐pollinated plants include grasses and sedges, making wind pollination more common in open environments. For instance, Monteiro *et al*. (2021) found that in the campos rupestres within the Cerrado biome, the frequency of wind pollination increased towards higher elevations where the frequency of grasses and sedges is higher. We are unaware of any water‐pollinated plants, although this may exist in the numerous water bodies and aquatic plants in the region. However, rain‐assisted self‐pollination can promote reproductive assurance in some orchids (e.g. Maciel *et al*., 2020).

### (2) Bees

Most Cerrado plant species are pollinated by bees (Fig. 3B; Martello *et al*., 2023; Luna *et al*., 2024). Bees constitute a speciose group in the biome, with an estimated number of species ranging between 1,200 and 1,500 (Raw, 2007). Pollination by this group is important even in open formations where wind pollination can be more common (Barbosa & Sazima, 2008). Several different groups of bees visit flowers, including diurnal and crepuscular species (Pacheco Filho *et al*., 2015; Araujo *et al*., 2020), although the roles of the latter are still underestimated. Although bees depend almost exclusively on flowers for survival and reproduction, they vary greatly in morphology and social organisation, in addition to the resources they collect in flowers (Willmer, 2011), with consequences on their interactions with plants. Nevertheless, large flight ranges can ensure cross‐pollination over distances up to 1 km, 5 km, and 30 km for small, medium, and large bees, respectively (Borges *et al*., 2020).

Medium and large bees are commonly solitary or subsocial, foraging individually for floral resources. These bees are diverse, belonging to different taxonomic groups (Andena, Santos & Noll, 2012; Pacheco Filho *et al*., 2015). As adults, most bees use nectar as an energy source for flight and mix pollen and other rewards, such as oil or nectar, to feed their larvae. They can also collect floral resources such as perfumes, oils, and resins that can be used for sexual attraction and nest building (Willmer, 2011). Although these bees can be more generalist or specialised regarding the flowers they visit, their guilds are commonly structured according to their resource use.

For example, carpenter bees of the genus *Xylocopa* (Xylocopini tribe) collect mainly pollen and nectar from different species (Araújo *et al*., 2021b), which they store in nests excavated in dead wood (Gerling, Velthuis & Hefetz, 1989). They pollinate many speciose and widespread plant groups, such as *Eriotheca* spp. (Malvaceae), *Kielmeyera* spp. (Calophyllaceae), several Fabaceae (e.g. *Cassia* spp., *Senna* spp., and *Chamaecrista* spp.), Solanaceae (e.g. *Solanum* spp., and *Cestrum* spp.) and Melastomataceae (e.g. *Rhynchanthera* spp., *Miconia* spp., and *Microlicia* spp.). In addition to some other families (Montesinos & Oliveira, 2015), plants from the latter three families commonly show a specialised mechanism where the pollen is held within poricidal anthers, and is only released through the apical pores after the flowers are vibrated, i.e. buzz‐pollination (Pacheco Filho *et al*., 2015). Such buzzing bees, including *Xylocopa* and other bee genera, e.g. *Bombus*, *Centris*, *Epicharis*, *Eulaema*, and *Oxaea*, can be pollinators of several dozen plants flowering in the same area (Pacheco Filho *et al*., 2015; Araújo *et al*., 2021b). Moreover, these bees can also use buzz‐pollination to improve pollen removal even from plants that do not bear poricidal anthers (Sazima & Sazima, 1989; Oliveira & Sazima, 1990; Sigrist & Sazima, 2004).

Other large bees, such as those of the Centridinae subfamily, are also solitary and collect floral resources from many plant species (Pacheco Filho *et al*., 2015). These bees also depend on oil to build their nests and feed the larvae, specialising in the flowers of the Malpighiaceae family (Gottsberger, 1986; Sigrist & Sazima, 2004). Such flowers usually have oil‐producing glands at the base of the calyx with stigmas covered with a thin membrane that pollinators must mechanically rupture to become receptive (Sigrist & Sazima, 2004). Thus, despite collecting nectar and pollen in flowers visited by other bees, the Centridinae is an example of how a guild can be more specialised in the use of a resource and how plants are obligatorily dependent on these pollinators (Pacheco Filho *et al*., 2015).

Many plants exploit the bee community, especially the larger solitary ones, by simulating the presence of fake rewards, i.e. have rewardless flowers. For example, floral mimicry is common in several orchids, such as in the genus *Cyrtopodium*, for which the Cerrado is the centre of diversity. Flowers probably mimic Malpighiaceae and depend on fortuitous visits from Centridinae bees (Fig. 3A; Maciel *et al*., 2020; Cardoso *et al*., 2023b). Although the colourful flowers and probably scent function as attractants, bees do not find oil or any other reward. Despite these plants receiving few visits because bees can learn that they are rewardless, even the occasional pollinations are sufficient to produce fruits with thousands of seeds (Maciel *et al*., 2020). In addition, after detecting a lack of resources, bees tend to avoid visiting flowers from the same individual, thus promoting cross‐pollination and high genetic diversity (Johnson & Schiestl, 2016).

Unlike other regions of the globe, large and solitary bees are equally or, in some contexts, more important than other bees for pollinating plants (Fig. 3B). However, small bees are more abundant and have several eusocial species, with organised nests and many non‐reproductive workers specialised in collecting floral resources. Sociality allows these bees to adopt mass recruitment that enables flower‐visiting workers to guide other bees in the nest, indicating the location and availability of higher‐rewarding floral resources (Willmer, 2011; Grüter, 2020). Besides the invasive *Apis mellifera*, the most reported social bees are the native stingless bee species of the tribe Meliponini (Pacheco Filho *et al*., 2015; Aguiar *et al*., 2024). They are also eusocial, and their recruitment seems to depend on scent marks left by workers on vegetation to guide nestmates (Grüter, 2020). Recruitment allows these eusocial bees to react rapidly to changes in resource supply in the environment. Accordingly, some plants show a ‘big bang’ mass‐flowering strategy, producing thousands of flowers for a short period (*sensu* Gentry, 1974; Oliveira & Gibbs, 2000). Eusocial bees may use these plants more effectively due to their recruitment capacity (Ramalho, 2004). Whether they are also the most effective pollinators is open to question, as such mass visitation may lead to frequent self and geitonogamous pollination. Although massive recruitment sometimes mobilises entire nests to forage on the same plant species, eusocial bees are not necessarily more specialised in resource use than solitary ones and can use more resource types over time (Rabeling *et al*., 2019; Santos *et al*., 2020; Pires *et al*., 2022). For instance, the social *Trigona spinipes* is the most frequent native floral visitor of the biome (Aguiar *et al*., 2024), and, together with other congeners, can act as pollinators. In addition, they can also be considered flower larcenists by consuming nectar and pollen without contacting the reproductive parts, often destroying anthers and other floral structures, thereby acting as robbers and thieves (Renner, 1983; Gottsberger, 1986; Araujo *et al*., 2020).

Some other speciose and abundant small‐ to medium‐sized bee species are not eusocial, including Euglossini, Halictidae, and some other groups (Pacheco Filho *et al*., 2015). Although they visit many groups of plants, their importance in pollination is less studied when compared to the bees mentioned above, even though they are still the exclusive pollinators of many species (e.g. Milet‐Pinheiro *et al*., 2015). Many of these bees, especially Euglossini, are more specialised in resource use, exploring flowers that offer perfumes, resins, and other rewards besides pollen and nectar (Milet‐Pinheiro *et al*., 2015; but see Pacheco Filho *et al*., 2015). However, they can behave similarly to other medium and large solitary bees, visiting many species and even performing buzz‐pollination. On the other hand, some plants may be associated with a wide taxonomic variety of bees. For instance, a study with the widespread *Matayba guianensis* (Sapindaceae) resulted in the sampling of 110 different bee species, all potential pollinators of this treelet, which have small and generalist flowers (Carvalho & Oliveira, 2010).

### (3) Beetles

Beetle pollination is relatively common, involving several Coleoptera families such as Chrysomelidae, Coccinellidae, Curculionidae, Erirhininae, Melolonthidae, and Nitidulidae (Silberbauer‐Gottsberger, 1990; Saravy, Marques & Schuchmann, 2021). Although there are several species of beetles, those of the genus *Cyclocephala* (Scarabaeidae: Dynastinae) constitute one of the most common groups, sometimes with a single species being the specialist pollinator of a plant (Saravy *et al*., 2021). Among other families, beetles commonly visit the flowers of Annonaceae and inflorescences of Araceae and Arecaceae (Fig. 3A; Gottsberger, 1986; Silberbauer‐Gottsberger, 1990; Saravy *et al*., 2021). These plants are usually protogynous (Fig. 2C, Table S2). Thus, stigma receptivity and pollen release occur separately on consecutive evenings and attract beetles *via* scent and colour cues. Some of these plants show large and highly specialised thermogenic floral chambers that increase the volatilisation of attractive compounds and simultaneously offer a safe and warm environment for the beetles inside (Gottsberger & Webber, 2018). Flowers of this group have a trap‐and‐release mechanism synchronised with the beetle circadian rhythm, maximising cross‐pollination (Gottsberger, 1999; Saravy *et al*., 2021). Beetles usually spend the night protected in these chambers while mating and feeding on pollen and nutritious tissue of the thickened floral whorls (Gottsberger, 1999; Gottsberger & Webber, 2018). Although these specialisations are common, some plants are pollinated by smaller beetles, with reduced and less specialised chambers, diurnal anthesis, and no thermogenesis (Gottsberger, 1999). Beetles are also important pollinators of some palm trees, including the emblematic *Mauritia flexuosa* (Arecaceae; Fig. 3A; Mendes *et al*., 2017), a dioecious palm that has also been described as wind‐pollinated (Fig. 3A; Rosa & Koptur, 2013) or even bee‐pollinated, ambiguous results as reported for other Neotropical palms (Silberbauer‐Gottsberger, 1990; Henderson, 2024).

### (4) Butterflies and moths

Although butterflies constitute a diverse group of insects with *ca*. 1,000 species in the Cerrado (Brown Jr & Mielke, 1967), they are regarded as less important pollinators, often overlapping with small bees and other insects in the flowers they visit (e.g. Machado & Oliveira, 2000; Sigrist *et al*., 2021; Martello *et al*., 2023, but see Pansarin & Ferreira, 2015).

Nocturnal moths are even more diverse, with at least 8,000 species estimated to occur in the biome (Camargo, 2001), and often acting as specialised pollinators of many species (Fig. 3; Oliveira, Gibbs & Barbosa, 2004). They frequently visit flowers with nocturnal anthesis that keep the nectar protected in tubes or spurs that only these long‐tongued insects can exploit. The size of the floral tube separates the small moths with short proboscises from those of the Sphingidae family with long proboscises (Oliveira *et al*., 2004). Hawkmoths can obtain nectar from long‐tubed flowers such as *Tocoyena formosa* (Rubiaceae), whose flowers may reach more than 15 cm in length (Oliveira *et al*., 2004). They can also explore flowers with open morphologies such as *Caryocar brasiliense* (Caryocaraceae), where they can be occasional pollinators (Gribel & Hay, 1993), or the specialised asclepiad *Schubertia grandiflora* (Apocynaceae) which despite short floral tubes ensures functional specialisation through its complex morphology (Amorim *et al*., 2022a). These large moths are long‐lived and can fly for long distances, possibly following the supply of floral resources throughout the different plant formations and regions of the biome (Amorim *et al*., 2009). This large flight range of moths mediates outcrossing. Notably, this group pollinates 21% of the 38 most widely distributed woody Cerrado species (Oliveira *et al*., 2004). For example, *Qualea grandiflora* (Vochysiaceae), the most extensively distributed tree in the biome (Ratter *et al*., 2003), is pollinated by Sphingidae moths that look for nectar accumulated in a spur formed in the calyx of flowers (Potascheff *et al*., 2020). Although less studied, nocturnal settling moths, especially from the Noctuidae family, also contribute to the high importance of moths as pollinators (Oliveira *et al*., 2004), sometimes sharing the plant species with other pollinators such as bees that visit the same flowers during the day (e.g. Maruyama, Amorim & Oliveira, 2010).

### (5) Other insects

Other insects, such as wasps and flies, can pollinate some plants. Although these insects frequently visit flowers, they often share with small bees the preference for generalist flowers, which are usually small, actinomorphic, and nectar‐rewarding (Fig. 3; Machado & Oliveira, 2000; Monteiro *et al*., 2021, 2025; Sigrist *et al*., 2021), such as in the case of *Piper* (Piperaceae) species (Valentin‐Silva, Batalha & Guimarães, 2021b). Such pollination by diverse insects has been less investigated in detail, probably due to their lower specialisation (see Moreira & Freitas, 2020). Nevertheless, they potentially contribute to supplementing plant pollination. For instance, 185 wasp species have been reported visiting 144 plant species in the Cerrado and mountainous grasslands of the Atlantic Forest (Monteiro *et al*., 2025). However, there are exceptions involving elaborate systems, such as the case of fly pollination *via* oviposition site mimicry in the Aristolochiaceae and some Orchidaceae (Cardoso *et al*., 2023a; Matallana‐Puerto *et al*., 2024, 2025) and wasp pollination in Asclepiadoideae (Apocynaceae) and *Ficus* (Moraceae) (Wiemer *et al*., 2012; Santos *et al*., 2022). There are also some cases involving pollination by other types of small insects, such as thrips in some generalist flowers (Oliveira, 1996a; Monteiro *et al*., 2021). These are the main pollinators of the widespread protogynous *Xylopia aromatica* (Annonaceae), in which they take shelter and feed on tissue and pollen released before they leave (Gottsberger, 1970, 1999; Saravy *et al*., 2021). Ant pollination has also been recorded in some Cerrado Apocynaceae, Eriocaulaceae, and Euphorbiaceae (Fig. 3A; Domingos‐Melo, Nadia & Machado, 2017; Del‐Claro *et al*., 2019; Faria *et al*., 2022).

### (6) Birds

Some plants are also pollinated by vertebrates (Fig. 3). As in other neotropical ecosystems, hummingbirds are one of the most conspicuous flower visitors. They usually hover in front of flowers during visits, allowing them to access nectar effectively (Ollerton, 2024). They are the pollinators of several species that typically show tubular flowers, bright colours, lack of scent as an attractant, and secretion of copious amounts of dilute nectar (Araújo, Sazima & Oliveira, 2013; Ferreira, Maruyama & Oliveira, 2016). These plants include herbs, shrubs, and some treelets, with hummingbird‐pollinated trees being rare (Fig. 3B; Araújo *et al*., 2013). Some hummingbirds may promote high outcrossing by showing traplining behaviour, following regular routes during their foraging among the patches of resources (Araújo, Barbosa & Oliveira, 2011a; Araújo *et al*., 2013; Maruyama, Justino & Oliveira, 2016), although some species may show territorial behaviour towards higher quality patches, which may restrict pollen flow (Maruyama *et al*., 2016). Territorial foraging may explain why Cerrado hummingbird‐pollinated plants are commonly self‐incompatible, unlike bird‐pollinated plants elsewhere (Ferreira *et al*., 2016; Maruyama *et al*., 2016).

Hummingbirds can effectively pollinate plants with specialised traits, such as some Ericaceae bearing poricidal anthers, where the hovering behaviour provides the vibration necessary to remove pollen grains effectively (Araújo, Farias & Oliveira, 2011b). Even though hummingbirds are well matched to ornithophilous flowers, they are opportunistic and visit any plants that offer accessible nectar to meet their energetic requirements (Araújo *et al*., 2013; Maruyama *et al*., 2013), and some non‐ornithophilous flowers have similar nectar volume, sugar concentration, and energy content to those more specialised ones (Araújo *et al*., 2021a). While these may not represent typically specialised hummingbird–flower interactions, hummingbirds may still contribute to the reproduction of non‐specialised plants in the region (Amorim *et al*., 2022b). A lower proportion of specialised hummingbird pollination is frequently reported in different habitats. Still, it can be more frequent in some formations, such as the rupestrian fields, potentially explained by their ability to provide long‐distance cross‐pollination (Monteiro *et al*., 2021).

Whereas hummingbirds are the predominant group of birds visiting flowers, some other birds also look for floral resources with greater or lesser intensity, sometimes sharing plants with hummingbirds (Pansarin, 2024). Other visiting birds typically do not hover in front of flowers; instead, they perch on the inflorescence or adjacent branches that can support their weight (Rocca & Sazima, 2010; Ollerton, 2024). Unlike perching birds from other parts of the world, those from the Cerrado are generalists and less dependent on plants (Rocca & Sazima, 2010). However, some plant species seem to depend almost exclusively on perching bird pollination, such as *Hortia brasiliana* (Rutaceae) (Fig. 3A; Barbosa, 1999). Other species that produce flowers close to the ground are also visited by birds, such as the root holoparasite *Langsdorffia hypogaea* (Balanophoraceae) (Santos *et al*., 2017).

### (7) Mammals

If during the day birds are the main vertebrates pollinating plants, during the night many flowers depend on bats for pollination. The first case described in detail was the emblematic *Lafoensia pacari* (Lythraceae) in a seminal paper by Sazima & Sazima (1975). They are key species, acting as pollinators and seed dispersers, sometimes of the same species (Sazima & Sazima, 1975). Several specialised nectarivorous bats can pollinate flowers by hovering in front of them during the visit like hummingbirds, while other bats hang on flowers (Sazima & Sazima, 1975; Gribel & Hay, 1993). They fly long distances and may also exhibit traplining behaviour, similar to hummingbirds (Sazima & Sazima, 1975), thereby promoting cross‐pollination. Bat‐pollinated flowers are usually white and secrete copious and dilute nectar, show a cornucopia flowering pattern, i.e. with a relatively long and massive flowering period (*sensu* Gentry, 1974), producing large amounts of nectar and attracting bats and other nocturnal visitors for several nights (Gribel & Hay, 1993; Gibbs, Oliveira & Bianchi, 1999).

Bats may share flowers with other visitors, including moths, bees, other mammals, and birds (Gribel & Hay, 1993; Vieira & Carvalho‐Okano, 1996; Araujo *et al*., 2020). For instance, bats were reported as relevant secondary pollinators of otherwise hummingbird‐pollinated *Psittacanthus robustus* (Loranthaceae), revealing their ecology as generalist nectar foragers (Diniz, Fischer & Aguiar, 2022). Further investigations may reveal other instances of such generalisation, increasing the importance of bats as pollinators.

Only a few trees seem to be adapted to pollination by non‐flying mammals. For instance, the tree *Pseudobombax tomentosum* (Malvaceae) includes opossums among its pollinators, with flowers showing adaptations such as large size, white colour, strong structures, upward orientation, a deep nectar chamber, and an erect pistil (Fig. 3A; Gribel, 1988).

## V. INTERACTION NETWORKS

**Fig. 4:**
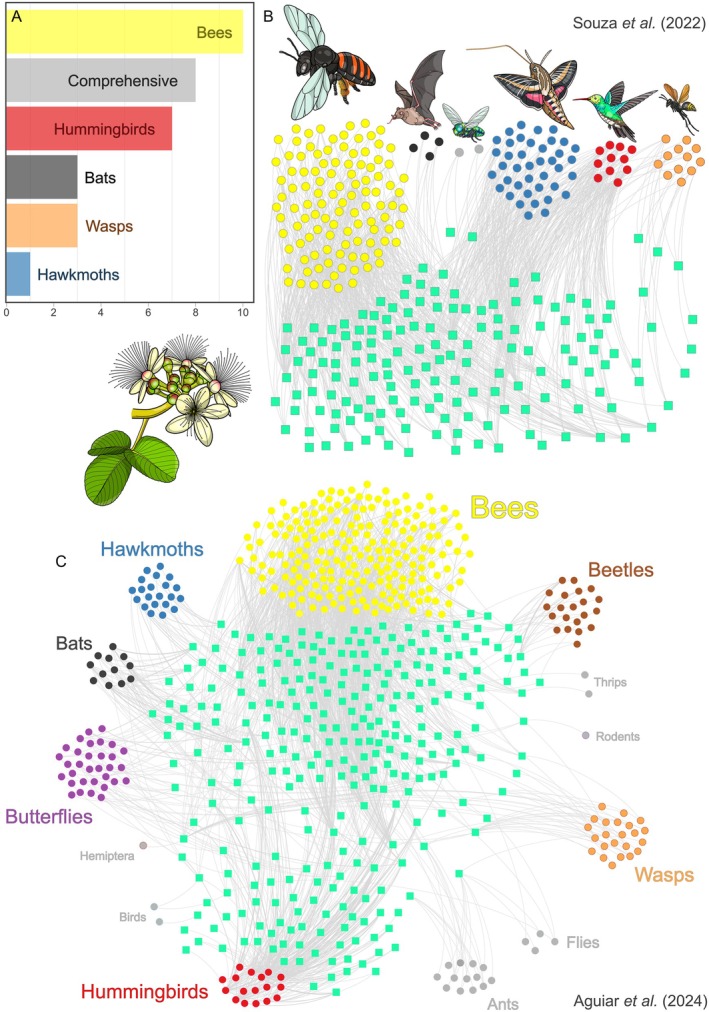
Cerrado plant–pollinator networks. (A) Number of studies conducted with each pollinator guild and with several guilds (i.e. comprehensive networks). (B) Comprehensive network of a single area and (C) meta‐network for the entire biome. The respective references of each network are indicated at the top and bottom right. Artwork credit: Lucas Kias.

The above‐described biotic pollination systems are embedded in a complex web of interactions reflecting many ecological processes structuring communities. Network approaches have boosted our understanding of how pollination interactions are organised in the Cerrado, synthesising the organisation of whole plant–pollinator communities or some specific functional pollinator groups (e.g. Souza *et al*., 2018, 2022; Rabeling *et al*., 2019). We reviewed the number of studies that reported pollination networks in the biome and gathered information from 32 research papers (Table S3; Appendix S1). Of these, 24 referred to specific functional groups (i.e. partial networks), with bees being the most frequently studied (10 studies), followed by hummingbirds (seven), wasps (three), bats (three), and hawkmoths (one) (Fig. 4A). Eight studies involved pollination interactions with different groups of animals (i.e. comprehensive networks) (Fig. 4A).

Comprehensive approaches are less often reported for tropical areas such as the Cerrado than in temperate regions of the world (Vizentin‐Bugoni *et al*., 2018). However, a recent study compiled night and day interactions from data gathered since the 1980s in a well‐studied Cerrado area and showed that the pollination network reflects the organisation of the pollinator guilds (Fig. 4B; Souza *et al*., 2022). It also showed that numerous important groups of plants are associated with different functional groups of pollinators, with some nocturnal flowering plants such as *Caryocar brasiliense* even acting as connectors between modules of diurnal and nocturnal pollination interactions (Souza *et al*., 2022). These patterns highlight how some plants are key species in maintaining different pollinator groups, even those not acting as primary pollinators. Another study took a broader approach, compiling all reported plant–pollinator interactions and building a meta‐network for the biome (Fig. 4C; Aguiar *et al*., 2024). It had 386 visitor species and 293 plant species (Aguiar *et al*., 2024), the latter corresponding to 2.4% of the Cerrado angiosperm flora. They found that bees were highly generalist visitors because they interacted with many species. On the other hand, hummingbirds and bats were more specialised, forming their own interaction modules (Aguiar *et al*., 2024). Their analyses also revealed that the mosaic of plant formations is connected by pollination interactions due to some generalist floral visitors associated with plants that occur in several formations (Aguiar *et al*., 2024; see Section VI).

Studies conducted with specific pollinator groups show how distinct ecological processes act on structuring pollination networks. For instance, studies conducted with both hawkmoths and hummingbirds showed that niche‐based processes linked to morphological matching among plants and pollinators are relatively more important than chance meetings governed by abundances in determining species interactions [i.e. neutrality of interactions (Maruyama *et al*., 2014; Sazatornil *et al*., 2016)]. This indicates that there is a clear fit between plant and pollinator morphology, where long‐tongued/billed pollinators mostly interact with equally specialised flowers, while those with short tongues/bills interact more often with short flowers (Maruyama *et al*., 2014; Sazatornil *et al*., 2016). Since niche‐based mechanisms are expected to be more important than neutrality when communities show higher trait/functional diversity (Vizentin‐Bugoni *et al*., 2018), such results support the high complexity of pollination interactions. Besides morphological fit, other factors, such as phenological differences across the mosaic vegetation, may also explain interactions. For bats, the occurrence of species across different plant formations was the best predictor of interactions, although phenological overlap and morphology also explain some aspects of network organisation (Diniz & Aguiar, 2023a,b). Interestingly, phenology and habitat occupation are also important drivers of hummingbird interactions (Maruyama *et al*., 2014), revealing common patterns across different pollination systems (see Section VI).

Considering the bees, which are the most studied group, their community forms species clusters based on the exploited plants, showing a nested structure with higher intra‐ and lower inter‐specific competition, thus allowing the coexistence of the many bee species (Andena *et al*., 2012). However, some plant species can be key resources, such as the oil‐producing *Byrsonima sericea* (Malpighiaceae), which promotes high niche overlap among several bees (Santos *et al*., 2020). Other studies focused on insect–flower interactions with an emphasis on the sociality of insects (Pires *et al*., 2022; Monteiro *et al*., 2025). Social bees and wasps occupied more central positions in the interaction networks than solitary species, showing that their generalised foraging behaviour potentially drives the entire community dynamics in pollination interactions.

Given the diversity of pollination systems and the diversity of species involved in these interactions, there is much to be learned about how pollination networks in this biome are structured. The limited evidence suggests that while the networks of plants with hummingbirds are fairly specialised (Maruyama *et al*., 2014), those with hawkmoths (Johnson *et al*., 2017), social wasps (Mello *et al*., 2011), and bats (Diniz & Aguiar, 2023a,b) are more generalised, indicating less partitioning of interactions. Given their ecological and evolutionary consequences, we need more specific pollination system studies to test whether different pollination systems show distinct structural patterns. Other factors relevant to the Cerrado, such as spatial heterogeneity, seasonality, and fire, may also influence the assemblage and networks of pollinators. Below, we discuss the effects of these.

## VI. SPATIOTEMPORAL CHANGES

Temporal changes occur even in preserved environments. Thus, the Cerrado mosaic formations are dynamic not only in space but also in time, which may affect pollination (Escobar‐Torrez *et al*., 2024). Landscape changes in the biome occur from small timescales over years or decades (Deus & Oliveira, 2016), to the scale of thousands of years (Oliveira‐Filho & Ratter, 2002; Escobar‐Torrez *et al*., 2024).

### (1) Seasonality and phenology

Annual cyclic changes are common and key in seasonal areas such as the Cerrado (Eiten, 1972; Klink *et al*., 2020). The reproductive phenology of plants is directly related to the resource supply to pollinators, which in turn are known to forage according to the temporal distribution of flowering species (Antonini & Martins, 2003). The marked seasonality creates cyclic and predictable climatic variations. Plants are synchronised to these environmental conditions, showing a seasonal and well‐defined flowering pattern at the population and community levels (Oliveira, 2008; Cardoso *et al*., 2022; Ferreira *et al*., 2023). At the population level, interspecific phenological differences allow the occupation of different temporal niches and can function as a mechanism to avoid competition for pollinators and to reduce heterospecific pollen deposition between phylogenetically related species (Oliveira & Sazima, 1990). However, there might be overlapping phenologies between closely related syntopic species that may avoid competition and hybridisation *via* mechanisms such as pollinator partitioning, morphological variations of flower organs, and incompatibility (Oliveira *et al*., 1992; Cardoso *et al*., 2022).

**Fig. 5:**
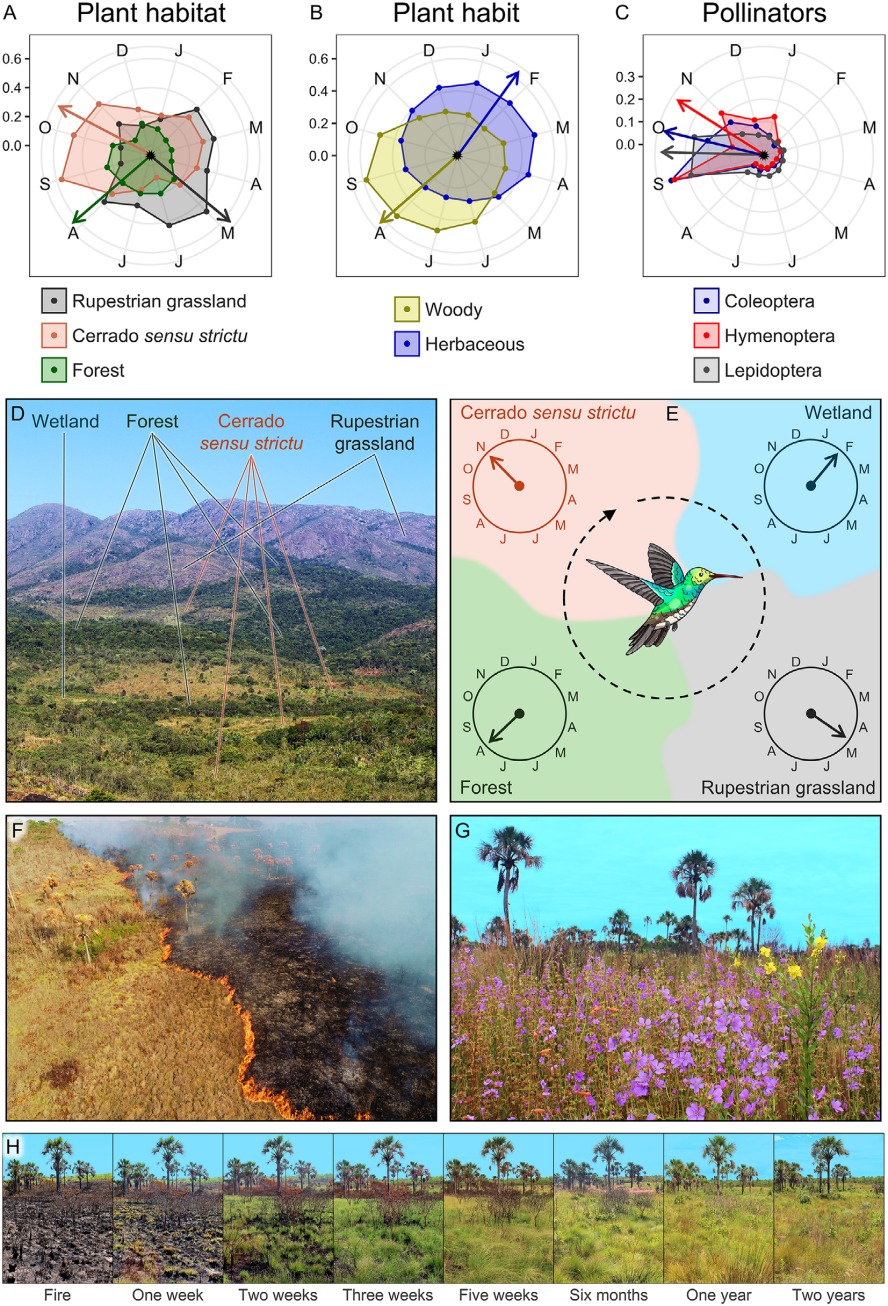
Spatiotemporal changes in the Cerrado and their influences on pollination. Radar charts illustrating temporal distribution patterns of plants and pollinators across wet (October–March) and dry (April–September) seasons. (A) Within‐habitat proportion of species flowering in a grassland–savanna–forest gradient under the same climatic conditions showing the differences across plant formations. (B) Proportion of species flowering in a cerrado *sensu* s*tricto* area showing the different flowering peaks of woody and herbaceous species. (C) Proportion of the abundance of insect individuals found in the same area showing the temporal distribution of the main insect pollinator groups. The arrows within the plots show the peaks of occurrence (i.e. the circular mean). Data for A, B, and C were extracted from Oliveira *et al*. (2021b), Batalha & Martins (2004), and Silva *et al*. (2011b), respectively (Appendix S4). (D) Vegetational mosaic of the Cerrado and (E) schematic representation of the habitat complementarity (based on hypothetical peaks) where patches of different plant formations have different diurnal or annual resource‐offering peaks that become complementary if pollinators move across the landscape. (F) Fire consuming a vereda vegetation and (G) post‐fire flowering in the same area showing the richness and abundance of flowers. (H) Long‐term time‐lapse demonstrating rapid vegetation recovery and resilience after fire.

At the plant community level, flowering patterns may offer insights into the resource availability for well‐defined pollinator guilds. Several community‐wide phenological studies have shown that the flowering patterns of plant communities are usually seasonal across several plant formations. For instance, dry grasslands, including the rupestrian fields, have their flowering peak during the wet season (October–March) or in the transition from wet‐to‐dry seasons (Fig. 5A, E; Appendix S4; Tannus, Assis & Morellato, 2007; Morellato, Camargo & Gressler, 2013; Le Stradic *et al*., 2018). Meanwhile, wet grasslands and vereda (Fig. 5E, G) peak during the dry‐to‐wet and wet seasons (Tannus *et al*., 2007; Luna *et al*., 2024). The plant community of denser cerrado *s.s*. formations (i.e. with a classic savanna structure) usually peaks between the end of the dry and the beginning to middle of the wet season (Fig. 5A, E; Batalha & Mantovani, 2000; Batalha & Martins, 2004; Morellato *et al*., 2013). Riparian and dry forests usually show peaks in reproductive activity during the wet season and, in some cases, during the end of dry and beginning of wet seasons (Fig. 5A, E; Neves *et al*., 2017). The temporal differences in resources available across the mosaic of plant formations suggest an annual habitat complementarity with pollinators able to acquire resources throughout the whole year if they move between the patches of resources (Fig. 5D, E; Araújo *et al*., 2011a; Maruyama *et al*., 2013; Aguiar *et al*., 2024), as also reported for other interactions such as between fruits and frugivorous birds (Maruyama *et al*., 2019).

It is important to highlight that although there is marked seasonality, there are always some species flowering at periods of the year outside the flowering activity peaks. In addition, phenologies also seem to be idiosyncratic across areas since some grasslands, cerrado *s.s*., and forests show flowering uniformity throughout the year (Neves *et al*., 2017; Le Stradic *et al*., 2018). Moreover, there might be differences within plant formations, usually related to plant habit and stratification, which can generate microhabitat complementarity. For instance, in cerrado *s.s*., the woody species have their flowering peak during the late dry and early wet season, while the herbaceous plants flower mainly during the late wet season (Fig. 5B; Batalha & Mantovani, 2000; Silva *et al*., 2021; Ferreira *et al*., 2023). In vereda formations, tree species provide their resources mainly during the rainy season, while smaller‐sized species (shrubs, herbs, and grasses) flower during the dry season (Luna *et al*., 2024). Such continuous resources offered by the plant community within habitats may sustain resident pollinators, such as social bees, which, in turn, provide effective pollination to plants at any time of the year.

However, some pollinator groups also show a seasonal pattern in their abundances that seems synchronised with the plants from which they obtain resources and *vice versa* across the landscapes (Gottsberger, 1986). This seems to be common, especially for invertebrates such as Coleoptera, Hymenoptera, and Lepidoptera (Fig. 5C; Antonini & Martins, 2003; Amorim *et al*., 2009; Silva, Frizzas & Oliveira, 2011b; Saravy *et al*., 2021). Despite these groups occurring throughout the year, their peaks coincide with those of cerrado *s.s*. flowering peaks, originally the most common vegetation type of the biome (Eiten, 1972; Borghetti *et al*., 2019). This highlights synchronisation and interdependence between plants and their potential pollinators over larger regional scales (Fig. 5A, C).

Although the reproductive phenology of plants and animals is relatively well understood, the temporal patterns of interactions have been less studied. The existing data show that although the proportion of each pollination system varies over time, bee pollination is the most important throughout the year (Luna *et al*., 2024). In addition, some pollination networks may offer evidence about the temporal arrangement of interactions. A comprehensive network study evaluated an area of dense grassland and another of vereda and showed that plant–pollinator networks show strong seasonal patterns (Souza *et al*., 2018). Interactions are more partitioned during the dry season when floral resource availability is lower, which can be attributed to potentially stronger competition for scarce resources (Souza *et al*., 2018). Alternatively, the network becomes less specialised during the resource‐abundant rainy season. A study conducted in another area also indicated that during the dry season, plants and bees perform fewer interactions with fewer partners, which led to a less robust interaction network in the dry than in the rainy season (Rabeling *et al*., 2019). Likewise, bat–flower networks seem to follow a similar trend, with interactions showing a higher tendency for specialisation and partitioning of interactions during the peak dry season than other periods of the year (Diniz & Aguiar, 2023a,b). Since floral resource partitioning affects ecosystem functioning, such temporal changes in interaction patterns are expected to affect pollination dynamics. Thus, seasonality seems to be a strong driver of pollination networks.

Altogether, the horizontal and vertical flowering variations at different scales in the Cerrado areas form a mosaic of resource supply throughout the year. Such year‐round distributions are particularly important to pollinators that need continuous energetic resources, such as bats, hummingbirds, and some solitary and social bees. Since these organisms have high energetic demands, they move between formations throughout the day and seasons, showing habitat complementarity across the vegetational mosaic (Fig. 5D, E; Araújo *et al*., 2011a; Maruyama *et al*., 2013; Aguiar *et al*., 2024). Thus, habitat connectivity and complementarity are essential processes in the dynamics of this biome. Interaction networks with different pollinator groups corroborate connectivity across the mosaic habitats. For instance, while different habitats are reflected in the network structural patterns of hummingbirds and bats (Maruyama *et al*., 2014, Diniz & Aguiar, 2023a,b), these are still connected through pollinator movement, especially as they follow seasonal differences in flowering patterns among habitats (Fig. 5D, E; Diniz & Aguiar, 2023a,b).

### (2) Fire and long‐term changes

Despite being seasonal and relatively predictable, the Cerrado is subject to supra‐annual changes. Seasonality causes the drying of accumulated biomass during the dry season, favouring the outbreaks of natural fires initiated by lightning (Fig. 5F; Oliveira‐Filho & Ratter, 2002). Fire is an important ecological factor that reduces biomass periodically and promotes eco‐evolutionary changes in the flora (Fig. 5G, H; Borghetti *et al*., 2019; Klink *et al*., 2020). For example, the Cerrado plant lineages started to diversify ten million years ago, coinciding with the rise of flammable C4 grasses (Simon *et al*., 2009; Borghetti *et al*., 2019). In addition to determining several structural, physiological, and ecological patterns of communities and populations (Borghetti *et al*., 2019; Klink *et al*., 2020; Fidelis & Zirondi, 2021), fire can also indirectly affect pollination and pollinators, an effect relatively well understood in other ecosystems (Brown *et al*., 2017). However, little research has been conducted linking the importance of fire to pollination and plant reproduction (Ballarin *et al*., 2024). Due to logistical difficulties, studies lack control groups with data before the fire or unburned areas, especially concerning post‐fire long‐term patterns (Ballarin *et al*., 2024).

From the little that is known, plants, pollinators, and their interactions are relatively resilient after natural fires (Fig. 5G, H; Baronio *et al*., 2021; Teixido *et al*., 2024; Trevizan *et al*., 2025), although some idiosyncratic effects may occur in space and time. Generally, fire immediately negatively impacts plants by destroying developing vegetative and reproductive structures (Borghetti *et al*., 2019). Some plant species do not flower after fire (Tunes *et al*., 2017; Fidelis & Zirondi, 2021). Others may invest more in vegetative than sexual reproduction (Borghetti *et al*., 2019). Some species may even die, especially those from forests that are not adapted to fire (Oliveira‐Filho & Ratter, 2002; Klink *et al*., 2020). However, it is well established that fire triggers the flowering of hundreds of species (Fig. 5G), with some fire‐dependent ones flowering only after a fire (Pilon *et al*., 2021; Fidelis & Zirondi, 2021). Thus, fire makes floral resources available to the community of pollinators, which respond by increasing visitation frequency (Carbone *et al*., 2024), boosting the outcrossing rate of plants (Franceschinelli & Bawa, 2005). A study with the widespread distylous *Palicourea rigida* (Rubiaceae) comparing contiguous burned *versus* unburned areas corroborated this resilience (Trevizan *et al*., 2025). Fire had both positive and negative effects, increasing fruit set in the short‐styled morph and slightly reducing some floral traits (e.g. corolla and anther sizes), respectively. However, it had no effect on display and rewarding traits or on between‐morph reciprocity. This resilience enables pollen flow within the fire‐affected area and across the mosaic of different fire histories, maintaining the polymorphism and the associated pollinators.

Considering phenology, the plant community is resilient and the flowering period does not change after fire (Valentin‐Silva *et al*., 2021a). However, fire may alter the temporal arrangement of floral resource provisioning to specific guilds, as shown by an experimental study using burned *versus* unburned plots (Tunes *et al*., 2017). While some resources become seasonal after fire, e.g. for flies, others become more evenly distributed throughout the year, such as for beetles, wasps, and medium and large bees. In addition, fire anticipates resource availability to some functional groups of pollinators (e.g. beetles, butterflies, flies, wasps, and small bees), and delays availability to others (e.g. medium and large bees) (Tunes *et al*., 2017). From a landscape‐level perspective, fire regimes can also play an important role since a repeated fire pattern can cause homogenisation of flowering phenologies across habitat patches and lead to temporal gaps in the supply of floral resources. Ferreira *et al*. (2023) investigated how the diversity of fire regimes, i.e. pyrodiversity, including at distinct periods of the year and different intensities, influences community flowering phenology. They found that pyrodiversity is important in promoting different flowering phenological patterns across patches. This habitat complementarity results in a diversified resource supply throughout the year that is important to pollinator maintenance (Ferreira *et al*., 2023).

Fire may negatively affect some members of the local community of pollinators, especially residents such as bee colonies in cavities and those that do not fly long distances or hide in aerial parts of plants or leaf litter. For instance, fire may decrease the richness of Euglossini bees in forest environments (Giehl *et al*., 2013). However, while air temperature during a fire in the Cerrado can exceed 800 °C, temperature variations in the soil below 5 cm depth are negligible (Miranda *et al*., 1993). Thus, fire probably has little effect on the community of pollinators that live and nest on the ground, such as some bees and beetles. Reflecting the adaptability of organisms to natural fires, the time since the last fire event does not affect plant and pollinator community diversities, flower functional traits, and the frequency of community‐wide plant‐life form, translating into similar interaction network metrics (Baronio *et al*., 2021; Teixido *et al*., 2024). These findings indicate that interactions among plants and pollinators are quite resilient to natural fire, which can be attributed to their adaptations and pollinator mobility, making exploring recently burned areas possible.

Finally, long‐term studies may show how pollination systems may change after fire. Pollination systems are generally resilient over several years without drastic changes (e.g. Amaral *et al*., 2013; Deus & Oliveira, 2016). A common trend is that fire triggers the flowering of grasses, temporarily increasing the richness and abundance of wind‐pollinated species. However, this effect may vary spatially, occurring in some plant formations but not others (Amaral *et al*., 2013). One study compared the distribution of pollination systems using a more detailed classification in two periods 20 years apart and found that the vegetation underwent a process of woody plant encroachment (i.e. vegetation densification; Deus & Oliveira, 2016). This is related to fire suppression and is a common pattern found in the biome, replacing grasslands with savanna and forest formations, with an increase in the richness of trees and shrubs (Borghetti *et al*., 2019; Deus & Oliveira, 2016). The ecological succession decreased herbaceous plant dominance and increased the diversity of pollination systems, including some specialised ones involving moth pollination and tubular flowers (Deus & Oliveira, 2016). These patterns can be explained by the new environment that allows the coexistence of plants from both open and closed plant formations, increasing diversity. However, continued fire suppression will probably make the vegetation denser, leading to the local extinction of species from more open plant formations (Durigan & Ratter, 2016; Gonçalves *et al*., 2021), probably decreasing the diversity of pollination systems in the long term.

Altogether, although fire can induce local changes, it maintains long‐term pollination systems in an area. Since fire effects are idiosyncratic, the diversity of landscapes under different stages of post‐fire succession and fire regimes may influence the pollination patterns. Thus, mosaics of periodically burned patches under different burning regimes are important to maintain the diversity of plant formations (Oliveira‐Filho & Ratter, 2002; Borghetti *et al*., 2019) and the diversity and dynamics of plant–pollinator systems.

## VII. ECOLOGICAL CONSEQUENCES

The characteristics of pollination and reproductive systems of Cerrado plants imply some ecological consequences that can help define conservation and management strategies to protect the biome. The first ecological consequence is the interdependence between plants and pollinators for survival and reproduction. Regarding conservation, our review demonstrates that most plants depend on pollination services for seed formation. Conversely, pollinators need floral resources for survival and reproduction. This strict interdependence seems characteristic of tropical environments and is a structuring factor of biological communities, especially in the tropics (Ollerton, 2017; Vizentin‐Bugoni *et al*., 2018). Although this interdependence rarely involves extremely specialised mutualisms between species or small groups, a decline in pollination services implies a cascade of consequences that would negatively affect ecosystem functioning. The main outcome of this type of organisation is maintenance of a high diversity of species and processes comparable to tropical forest formations (Fig. 3B; Myers *et al*., 2000; Strassburg *et al*., 2017). There is a growing consensus that pollination effects in ecosystems can go beyond ensuring plant reproduction, influencing other unrelated members of the community and biogeochemical cycles (Ollerton, 2021; Cardoso *et al*., 2023b). Thus, the interaction between plants and pollinators is one factor explaining the maintenance of biodiversity (Ollerton, 2017; Wei *et al*., 2021), which also applies to the Cerrado.

A second ecological consequence related to pollination systems is the complementarity of habitats in resource utilisation by pollinators at the landscape level, encompassing not only floral resources but also other essential elements, such as the availability of nesting and resting sites. The diversity of plant formations under different post‐fire stages of ecological succession and different fire regime histories is reflected in a mosaic of resource availability that pollinators exploit throughout time (Oliveira, 2008; Ferreira *et al*., 2023; Martello *et al*., 2023). The daily and annual movements across patches of resources ensure pollinator maintenance while increasing gene flow and genetic variability, enabling plant reproduction (Oliveira, 2008; Aguiar *et al*., 2024). For instance, data on pollination by hummingbirds indicate that specific patches of savannas and forests do not offer sufficient floral resources to maintain resident populations (Araújo *et al*., 2013; Maruyama *et al*., 2013). Thus, hummingbirds must explore larger areas and several plant formations to meet their daily energy needs. As another example, although *Xylocopa* spp. and other bees reproduce throughout the year (Araújo, Lourenço & Raw, 2016), the availability of floral resources within plant formations changes considerably (see Section VI). Thus, these large bees exploit floral resources in different plant formations throughout the year, and the pollen used in nest provisioning seems to follow the flowering patterns (Araújo *et al*., 2021b). As the vegetation is heterogeneous and there are flowering differences across space and time, the landscape constitutes a mosaic of floral resources that pollinators exploit by adjusting their behaviour. This habitat complementarity implies complexity in the organisation of pollination systems and underscores the need to conserve different plant formations for pollinator maintenance at the landscape level (Silva *et al*., 2021; Aguiar *et al*., 2024).

Finally, the organisation into guilds probably has an ecological consequence of promoting relative redundancy of pollination systems across space and time. This pre‐adaptation to environmental heterogeneity and differences in diversity among areas (Ratter *et al*., 2003; Bridgewater *et al*., 2004; Martello *et al*., 2023) has likely enabled plants and pollinators to occupy large regions on a continental scale. For example, the carpenter bee *Xylocopa frontalis* occurs from Argentina to Mexico (Moure & Melo, 2023), probably adjusting to local floristic and phenological changes of the plants it uses for foraging and nest provisioning. This flexibility makes it one of Brazil's species with the highest number of interactions with plants (Oliveira *et al*., 2024b). As another example, large moths are highly mobile and explore floral resources across extensive areas (Amorim *et al*., 2009). The hawkmoth assemblage of the Cerrado shares species with different bordering biomes, such as humid forests and dry Caatinga and Chaco formations, functioning as a ‘crossroads’ in the regional movement of those animals (Amorim *et al*., 2009). Due to the high plant β‐diversity across areas, pollinators can adjust to local resource availability.

In agreement, some studies in different areas of the Cerrado have shown that the turnover of plant species used by hummingbirds increases according to geographic distance between areas (Machado & Oliveira, 2015). Conversely, hummingbird species change less across areas. The same idea applies to some dominant plant species. For instance, some outcrossing *Qualea* spp. and *Byrsonima* spp. have a widespread distribution (Ratter *et al*., 2003; Bridgewater *et al*., 2004). Even with specialised pollination mechanisms involving moths and oil‐collecting bees, respectively (Gottsberger, 1986; Sigrist & Sazima, 2004; Oliveira *et al*., 2004; Potascheff *et al*., 2020), these species thrive by exploiting the local faunas of pollinators. As networks can rewire according to the available mutualists, this can confer relative resilience to pollination systems in the face of many imminent threats. Although the structure in guilds can help to mitigate negative effects, the signs of impacts are already evident and call for urgent decision‐making.

## VIII. FUTURE RESEARCH DIRECTIONS

In addition to discussing the available information and highlighting the processes and patterns underlying pollination in the Cerrado, this review also serves as a starting point to guide future research agendas to expand the scope of basic and applied research. These should increase taxonomic coverage and the body of knowledge. For instance, only 2.4% of plant species in the Cerrado were represented in the biome‐wide plant–pollinator meta‐network (Aguiar *et al*., 2024), indicating that only a small portion of the immense diversity has received attention, underscoring that we are still far from achieving comprehensive sampling. This reinforces the importance of more field studies, in addition to syntheses that encompass a broad range of available data, for example, considering grey literature and unpublished data consolidated through collaborative researcher networks. In parallel, modern techniques should be incorporated to confirm patterns and address open gaps. Some targets of future research are listed below.

(1) Studies on the natural history of plants and pollinators at both the population and community levels remain needed. This will provide a more comprehensive view of reproductive systems, including asexual and sexual reproduction, incompatibility mechanisms, floral systems, pollinator guilds, interaction networks, and phenological patterns. Contemporary methods, including acoustic, visual, and chemical ecology (e.g. Kantsa *et al*., 2019), and DNA metabarcoding (e.g. Martins *et al*., 2023) should be employed to uncover hitherto unknown pollination processes.
(2) To understand better the influence of fire on pollination using experimental designs with appropriate controls, including non‐burned areas and different periods since burning (Ballarin *et al*., 2024; e.g. Trevizan *et al*., 2025). These approaches will be important for understanding the future of pollination in the face of increasing anthropogenic fires and will also provide evidence to support controlled burns.
(3) Use modern ecological niche modelling methods (Thuiller, 2024) to assess the current and future distributions of sexual and asexual taxa. Specifically, it will be interesting to test the geographic parthenogenesis hypothesis to determine whether apomictic groups will have an advantage in the future, under climate change and pollinator shortage scenarios.
(4) Conduct more detailed investigations into the prevalence of habitat complementarity. This can be addressed using classical palynological studies involving pollen from different sources, such as larval food in brood cells, pollinators' bodies, or heterospecific pollen deposited onto stigmas (e.g. Araújo *et al*., 2021b). In addition, modern techniques can provide strong spatial evidence, such as pollen tracking using quantum dots and DNA metabarcoding, and pollinator tracking *via* radiofrequency identification microsensors (e.g. Minnaar & Anderson, 2019; Nunes‐Silva *et al*., 2020). The knowledge generated will support strategies to mitigate landscape‐scale impacts and promote conservation initiatives encompassing the entire Cerrado vegetation mosaic.
(5) More applied studies that address the sustainability of pollination ecosystem services in maintaining productive landscapes, including native and cultivated plants used by humans (e.g. Machado *et al*., 2024; Altomare *et al*., 2025). In addition to the intrinsic value of maintaining ecosystem integrity, as addressed in our review, this approach will highlight the economic and cultural values of pollination, creating general awareness.
(6) Considering the importance of the Cerrado as Brazil's main agricultural frontier and a leading crop‐producing region globally, another applied topic consists of research evaluating the current and future threats to pollination in the biome. Key threats for investigation include land‐use changes (e.g. habitat reduction and fragmentation), pollution (e.g. pesticides and herbicides), global warming, and the introduction of non‐native plant and pollinator species (e.g. Martello *et al*., 2023). Specifically, we should explore how these threats affect the taxonomic and functional diversity of plants and pollinators, the sexual reproduction of plants, interaction networks, and phenologies.

## IX. CONCLUSIONS

(1) Over the last five decades, much natural history knowledge has been produced about the Cerrado and the organisms that inhabit it. Additionally, recent techniques involving molecular markers, ecological networks, and fossil pollen records have provided perspectives on the reproductive biology of plants and their interactions with pollinators. This hard and valuable work of many researchers has changed how we see plant reproduction and pollination systems, from simple and asexual mechanisms in the mid‐20th century to complex and intricate processes.
(2) Our review clarifies the processes behind Cerrado biodiversity and underscores that pollination is a key interaction that needs to be conserved at the landscape scale. Both plants and pollinators are adapted to seasonality and fire, the key natural factors of the biome that determine the structuring of complex landscapes. Plant communities usually flower throughout the year, but different flowering peaks in plant formations characterise habitat complementarity. This implies that the mosaic of different plant formation types supports pollinators continuously, ensuring the functioning of pollination systems that provide essential ecosystem services for human health and the economy.
(3) The intense destruction of this biome takes place on multiple fronts at rates faster than those of scientific advancement and conservation. The rapid environmental degradation (Fig. 1F) poses a threat that may lead to the extinction of some pollination systems even before we know them. Our review unveils patterns in plant reproduction and pollination biology for the Cerrado, synthesising hitherto underappreciated patterns relevant to the conservation of this megadiverse biome.
(4) The core insight of this review is the intricate dependence between plants and pollinators, structured across the landscape and seasons. Thus, the simultaneous conservation and restoration of various plant formations across the landscape mosaic is essential to ensure the habitat complementarity necessary to safeguard pollinator guilds and the ecosystem functions and benefits they provide, such as the maintenance of native plants and crop pollination. While some formations, such as forests, riverbanks, and water springs, are protected by Brazilian legislation, these in isolation are insufficient to maintain high pollinator diversity, requiring the simultaneous maintenance of the neglected grassland and savanna formations.
(5) Without integrated protection of complex landscapes interconnected by pollinators, ecosystem functions will certainly decline because the reduction in pollinator populations will lead to insufficient reproduction of plants and the eventual collapse of their populations. Indeed, this seems to be happening already. Due to the interdependency between plants and pollinators, the future scenario involves coextinctions and biotic homogenisation. Hence, the future of the Cerrado lies in an effective conservation strategy that values not only species diversity but also the diversity of habitats that characterise this complex ecosystem.

## Supporting information


**Appendix S1.** Methodology of systematic reviews.

**Appendix S2.** Cerrado maps and characterisation.

**Appendix S3.** Detailed description of the Costa Rican rainforest.

**Appendix S4.** Phenology figures.

**Table S1.** Results from the literature survey on apomictic systems.

**Table S2.** Results from the literature survey on floral systems.

**Table S3.** Results from the literature survey on plant–pollinator networks.
