# Supplementary material for: Pollination and plant reproduction in the Cerrado, the world's most biodiverse savanna
Source: Biol Rev Camb Philos Soc. 2025 Sep 16;101(1):74–105. doi: 10.1111/brv.70073 (PMC12783448; doi:10.1111/brv.70073)
Supplement: Supplementary file 1 — Appendix S1. Methodology of systematic reviews. [file BRV-101-74-s002.docx]

**Appendix S1. Methodology of systematic reviews**

**(1) Apomixis survey**

We investigated the number of Cerrado species reported in the literature as having an apomictic system (*sensu* Cardoso *et al*., 2018). To increase our reach, we utilized *Google Scholar* (scholar.google.com.br), as it returns grey literature. The last search was on November 27, 2024. To return results from the biome, we specified the terms “*Cerrado*” and “*Brazilian savanna*”. We conducted searches in both English and Portuguese, following the respective structures: (1) "*apomixis*" OR "*agamospermy*" AND "*Cerrado*" OR "*Brazilian savann*a"; and (2) "*apomixia*" OR "a*gamospermia*" AND "*Cerrado*" OR "*savana brasileira*" (Table S1). The references returned were read to investigate (1) if the plant occurrence matched the Cerrado biome distribution, (2) the name of the apomictic species considered, and (3) the type of apomixis. We checked the validity of the returned species names using the GBIF (Global Biodiversity Information Facility) taxonomic database (https://www.gbif.org/tools/species-lookup). Species that were not found in the GBIF database were checked against the Reflora database (https://reflora.jbrj.gov.br). If two or more studies reported the same species, it was considered only once in the final counts.

**(2) Floral systems survey**

We investigated the number of species reported in the literature as having the following floral system types (*sensu* Cardoso *et al*., 2018): distyly, stylar dimorphism, enantiostyly, heteranthery, dichogamy, and cleistogamy. We used *Google Scholar* with the last search on June 28, 2024. We followed the same protocol described above for apomictic systems. For instance, search terms for *distyly* were: (1) "*distyly*" AND "*Cerrado*" OR "*Brazilian savanna*" (for English); and (2) "*distilia*" AND "*Cerrado*" OR "*savana brasileira*" (for Portuguese) (Table S2). After checking the returned results in both GBIF and Reflora databases, two species did not match and were excluded from further analysis (Table S2).

To demonstrate visually which families are related to each of the floral systems, we conducted a correspondence analysis using the R-package *FactoMineR* version 2.11 (Lê, Josse & Husson, 2008). This procedure allows the graphical display of columns and rows in a low-dimensional space, thus revealing relationships among variables (Sourial *et al*., 2010). We did not include families and floral systems (stylar dimorphism) with few occurrences (≤ 5 species). Analyses were carried out in R software version 4.4.1 (R Core Team, 2024).

**(3) Interaction network survey**

We investigated the plant–pollinator networks in the Cerrado by surveying the number of studies published. We used *Google Scholar* with the last search on August 21, 2024. We used the following search structures to access studies in English and Portuguese, respectively: (1) "*pollination*" AND “*network$*” AND "*Cerrado*" OR "*Brazilian savanna*"; and (2) "*polinização*" “*rede$*” AND "*Cerrado*" OR "*savana brasileira*" (Table S3). We selected only studies conducted in natural environments, excluding those focusing on crops and urban areas. We considered only original articles, excluding unpublished literature (e.g. master’s and doctoral theses), as these could overestimate the final numbers by accounting for the same networks originating from different sources. We selected only studies that sampled plant–pollinator interactions at the community scale, involving different species for both pollinators and plants simultaneously. Studies encompassing different guilds of pollinators are referred to as comprehensive networks, while those focusing on specific functional groups are referred to as partial networks (*sensu* Vizentin-Bugoni *et al*., 2018).
