# Supplementary material for: Pollination and plant reproduction in the Cerrado, the world's most biodiverse savanna
Source: Biol Rev Camb Philos Soc. 2025 Sep 16;101(1):74–105. doi: 10.1111/brv.70073 (PMC12783448; doi:10.1111/brv.70073)
Supplement: Supplementary file 2 — Appendix S2. Cerrado maps and characterisation. [file BRV-101-74-s004.docx]

**Appendix S2. Cerrado maps and characterisation**

We used the last/updated version of the Cerrado geographic boundaries introduced by Vieira *et al*. (2022), which delimits an area of 2,230,841 km^2^. Bioclimatic data, including mean annual temperature, temperature seasonality, annual precipitation, and precipitation seasonality, were extracted from *WorldClim* v. 2.1 at 2.5 arc-mins resolution (*ca.* 4.1 km) based on historical climate data from 1970 to 2000 (Fick & Hijmans, 2017). The climate diagram was built following Walter & Lieth (1967). We extracted land cover data from the *MapBiomas* database v. 7.1 (Souza *et al.*, 2020) on *Google Earth Engine* (Gorelick *et al.,* 2017) on August 25, 2023. Land cover raster was resampled to match the following categories in *ArcGIS Pro* v. 3.1: forest (MapBiomas classes: forest formation, mangrove, wooded sandbank vegetation), savanna (savanna formation, wetland), grassland (grassland, salt flat, rocky outcrop, herbaceous sandbank vegetation, other non-forest formations), degraded (pasture, agriculture, temporary crop, soybean, sugar cane, rice, cotton, other temporary crops, perennial crop, coffee, citrus, other perennial crops, forest plantation, mosaic of uses, urban area, others), water, and non-observed.
