# Supplementary material for: Pollination and plant reproduction in the Cerrado, the world's most biodiverse savanna
Source: Biol Rev Camb Philos Soc. 2025 Sep 16;101(1):74–105. doi: 10.1111/brv.70073 (PMC12783448; doi:10.1111/brv.70073)
Supplement: Supplementary file 3 — Appendix S3. Detailed description of the Costa Rican rainforest. [file BRV-101-74-s006.docx]

**Appendix S3. Detailed description of the Costa Rican rainforest**

Since this forest is located outside the Cerrado Biome, we provide additional information. La Selva Biological Station is located within a tropical wet forest in the Sarapiquí canton of Heredia province, Costa Rica (10º26’ N, 84º00’ W). The reserve covers 1,536 hectares, mainly located in natural boundaries provided by the Peje River to the west and the Sábalo-Esquina creeks to the east. The forest is evergreen and characterized by a high daily temperature variation (6–12°C) relative to a low annual variation in mean temperatures (< 3°C), and receives an average of 4,000 mm of rainfall annually (McDade & Hartshorn, 1994). The overall biological richness of La Selva is remarkable among tropical wet forests, supporting a diverse and intact biodiversity. The area has a diverse vascular flora typical of a tropical rainforest, including many tree species, lianas, epiphytes, and broad-leaved monocots (Hartshorn & Hammel, 1994). Additionally, the fauna consists of many organisms, including pollinators, such as birds, bats, bees, moths, butterflies, and other invertebrates (McDade & Hartshorn, 1994).
