# Supplementary material for: Pollination and plant reproduction in the Cerrado, the world's most biodiverse savanna
Source: Biol Rev Camb Philos Soc. 2025 Sep 16;101(1):74–105. doi: 10.1111/brv.70073 (PMC12783448; doi:10.1111/brv.70073)
Supplement: Supplementary file 4 — Appendix S4. Phenology figures. [file BRV-101-74-s007.docx]

**Appendix S4. Phenology figures**

To obtain the flowering patterns for the different Cerrado formations, we based on Oliveira *et al*. (2021*b*) and their respective raw data (https://figshare.com/s/6d463a3ae0716efea08a). Data on flowering intensity were transformed into presence or absence. We then calculated the proportion of species flowering per month within each habitat. We removed one forest species (*Marlierea clausseniana*) since it had a missing observation for one month. We used *ImageJ* (Schneider, Rasband & Eliceiri, 2012) to extract data referring to the proportion of species flowering according to plant habit from Batalha & Martins (2004). To obtain the proportion of insect individuals for each order, we used raw data from Silva *et al*. (2011*b*), kindly made available by one of the authors (Charles M. de Oliveira).
